# The effect of modelling parameters in the development and validation of knee joint models on ligament mechanics: A systematic review

**DOI:** 10.1371/journal.pone.0262684

**Published:** 2022-01-27

**Authors:** Sara Sadat Farshidfar, Joseph Cadman, Danny Deng, Richard Appleyard, Danè Dabirrahmani

**Affiliations:** 1 Macquarie Medical School, Faculty of Medicine, Health and Human Sciences, Macquarie University, Sydney, New South Wales, Australia; 2 Sydney Medical School, Faculty of Medicine and Health, The University of Sydney, Sydney, New South Wales, Australia; Tokai University, JAPAN

## Abstract

**Background:**

The ligaments in the knee are prone to injury especially during dynamic activities. The resulting instability can have a profound impact on a patient’s daily activities and functional capacity. Musculoskeletal knee modelling provides a non-invasive tool for investigating ligament force-strain behaviour in various dynamic scenarios, as well as potentially complementing existing pre-planning tools to optimise surgical reconstructions. However, despite the development and validation of many musculoskeletal knee models, the effect of modelling parameters on ligament mechanics has not yet been systematically reviewed.

**Objectives:**

This systematic review aimed to investigate the results of the most recent studies using musculoskeletal modelling techniques to create models of the native knee joint, focusing on ligament mechanics and modelling parameters in various simulated movements.

**Data sources:**

PubMed, ScienceDirect, Google Scholar, and IEEE Xplore.

**Eligibility criteria for selecting studies:**

Databases were searched for articles containing any numerical ligament strain or force data on the intact, ACL-deficient, PCL-deficient, or lateral extra-articular reconstructed (LER) knee joints. The studies had to derive these results from musculoskeletal modelling methods. The dates of the publications were between 1 January 1995 and 30 November 2021.

**Method:**

A customised data extraction form was created to extract each selected study’s critical musculoskeletal model development parameters. Specific parameters of the musculoskeletal knee model development used in each eligible study were independently extracted, including the (1) musculoskeletal model definition (i.e., software used for modelling, knee type, source of geometry, the inclusion of cartilage and menisci, and articulating joints and joint boundary conditions (i.e., number of degrees of freedom (DoF), subjects, type of activity, collected data and type of simulation)), (2) specifically ligaments modelling techniques (i.e., ligament bundles, attachment points, pathway, wrapping surfaces and ligament material properties such as stiffness and reference length), (3) sensitivity analysis, (4) validation approaches, (5) predicted ligament mechanics (i.e., force, length or strain) and (6) clinical applications if available. The eligible papers were then discussed quantitatively and qualitatively with respect to the above parameters.

**Results and discussion:**

From the 1004 articles retrieved by the initial electronic search, only 25 met all inclusion criteria. The results obtained by aggregating data reported in the eligible studies indicate that considerable variability in the predicted ligament mechanics is caused by differences in geometry, boundary conditions and ligament modelling parameters.

**Conclusion:**

This systematic review revealed that there is currently a lack of consensus on knee ligament mechanics. Despite this lack of consensus, some papers highlight the potential of developing translational tools using musculoskeletal modelling. Greater consistency in model design, incorporation of sensitivity assessment of the model outcomes and more rigorous validation methods should lead to better agreement in predictions for ligament mechanics between studies. The resulting confidence in the musculoskeletal model outputs may lead to the development of clinical tools that could be used for patient-specific treatments.

## Introduction

The knee joint is a crucial, load-bearing joint with complex interactions between articular surfaces, cartilage, tendons, and ligaments. The ligaments in the knee are particularly prone to injury and rupture during dynamic activities, resulting in increased knee instability with profound impacts on patients’ daily living and functional capacities [1, 2]. Musculoskeletal knee modelling is a valuable tool for non-invasively investigating ligament force-strain behaviour under various applied boundary conditions to identify potential high-risk movements which will disrupt ligament integrity and produce instability [1]. The authors also believe that these techniques could provide an effective tool to pre-plan surgical reconstruction techniques.

Several different approaches to predicting ligament loads have been reported, with consideration to geometric input (e.g., joint contact surfaces or ligament insertion and bundling) and ligament material properties (e.g., stiffness and length), as well as other considerations, such as the degrees of freedom and type of activity [3–6]. These parameters all appear to be important in predicting knee joint kinematics and ligament mechanics and can be incorporated into musculoskeletal modelling [7, 8].

However, as the number of these parameters is increased to more accurately model natural knee motion, it becomes increasingly difficult to determine which parameters have the greatest overall effect on predicting ligament behaviour [3]. Additionally, it becomes difficult to perform sensitivity analyses on these parameters, given there is no standard approach, as the process depends on how the knee is modelled and what motion is prescribed; Different models will invariably emphasise different parameters resulting in outcomes unique to the conditions and movements defined by the original investigator [9]. The lack of consistency in modelling methodologies combined with the differences in kinematic and kinetic data acquisition techniques between research groups, makes reproducibility of results a serious limitation to the potential for translation of musculoskeletal modelling into broader clinical applications.

This systematic review aims to evaluate existing musculoskeletal models of the knee joint, focusing on modelling parameters used in various simulated movements. Furthermore, this review aims to identify the parameters with the highest impact on ligament mechanics, with the goal of informing researchers how to begin to standardise their models to achieve greater reliability. Only with standardise/validated models will musculoskeletal modelling be accepted as a clinical tool.

## Materials and methods

A systematic review was performed according to the PRISMA (Preferred Reporting Items for Systematic Reviews and Meta-Analyses) guidelines [10]. This study aimed to investigate the results of the most recent studies using musculoskeletal modelling techniques to create models of the knee joint, including the ligaments and reported the ligament forces/strains.

### Literature search and study selection

The following databases were searched: PubMed, ScienceDirect, Google Scholar, and IEEE Xplore. Different combinations of the terms “Knee”, “Model”, “Musculoskeletal”, “Ligament”, “load”, ‘‘force”, “tension “, “length”, ‘‘strain”, “elongation” and ‘‘lengthening” was used. The reference lists of identified original articles were also searched manually for relevant articles. The search was limited to English and full-text articles only, and all duplicate papers were first removed. A team of two reviewers (SSF and DD) then independently screened the titles and abstracts of the remaining papers for eligibility. Disagreements between reviewers were resolved by consensus.

#### Eligibility criteria

To avoid selection bias, inclusion and exclusion criteria were decided on before the review; these were applied once all articles were retrieved. Inclusion criteria were:

➢ The study focused on musculoskeletal knee modelling.
➢ The study modelled both the femur and tibia and the knee joint’s ligaments.
➢ The study modelled the human knee joint, and where required, participants or cadaveric specimens must be human subjects (males or females) of any age.
➢ The study modelled the intact knee joint [excluding total knee replacements (TKRs), uni-compartmental knee replacements (UKRs) and high tibial osteotomies (HTOs)].
➢ The study modelled the intact, ACL-deficient, PCL-deficient, or lateral extra-articular reconstructed (LER) knee joints.
➢ The study reported ligament mechanics (e.g., force, strain, or elongation)
➢ The study was published between 1 January 1995 and 30 November 2021.

Studies exclusively based on finite element modelling were excluded, as were non-English and non-peer-reviewed articles.

### Data extractions

A customised data extraction form was created to extract each selected study’s critical musculoskeletal model development parameters. One author (SSF) undertook the data extraction, and the other (DD) checked the final table to ensure reliability. Specific parameters of the musculoskeletal knee model used in each eligible study were independently extracted, including the (1) musculoskeletal model characteristics (i.e., software used for modelling, knee type, source of geometry, the inclusion of cartilage and menisci, and articulating joints and joint boundary conditions (i.e., number of degrees of freedom (DoF), subjects, type of activity, collected data and type of simulation)), (2) specifically ligaments modelling techniques (i.e., ligament bundles, attachment points, pathway, wrapping surfaces and ligament material properties such as stiffness and reference length), (3) sensitivity analysis, (4) validation approaches, (5) predicted ligament mechanics (i.e., force, length or strain) and (6) clinical applications if available.

These items were chosen to overview each selected study’s modelling and validation techniques and the presented sensitivity assessment, ligament mechanics, and associated results. In the absence of the published numerical data, data was obtained from the graphs. When a method was partially described in the original study, detailed information was retrieved from the authors’ references and previous works to provide comparable data across the selected studies.

### Quality assessment

A customised checklist consisting of 16 appraisal questions was developed based on previous reviews in computational modelling of the human motion for prediction modelling studies with clinical/translational outcomes [11] and human motion analysis [12, 13], assessing the quality of the selected studies. Each question was rated two (satisfying description or justification), one (limited details) or zero (no information). The 16-item quality checklist used in this review is listed in in Table 1.

**Table 1:** The checklist used for quality assessment of the included publications in the systematic review.

**Questions**
1. Are the research objectives clearly stated? (Objectives)
2. Is the study design clearly described? (Study Design)
3. Is the scientific context clearly explained? (Study Design)
4. Were participants or subjects selected appropriately and their characteristics adequately described? (Study Design)
5. Is the general musculoskeletal modelling technique adequately described? (Modelling Technique)
6. Is the ligament modelling method adequately described? (Modelling Technique)
7. Did the researchers assess the sensitivity of the model outputs on ligament model parameters? (Modelling Technique)
8. Were the loading and boundary conditions correctly defined based on the type of motion tasks? (Movement Tasks)
9. Were the simulation methods clearly described? (Simulation)
10. Was the validation methodology and results clearly described? (Validation)
11. Were the statistical methods justified and appropriately described (other than descriptive statistics)? (Statistics)
12. Were the primary outcomes clearly stated and supported by the results? (Results)
13. Did other literature support the key findings? (Key Findings)
14. Does the study add value to academia or the clinical community? (Key Findings)
15. Were the limitations of the study clearly described? (Limitations)
16. Were conclusions drawn from the study clearly stated? (Conclusion)

Each study was evaluated independently by the two authors (SSF, DD). The original article was checked to ensure the correct rated scores, and the authors found a consensus in case of discrepancy.

## Results

### Search yield

**Fig 1:**
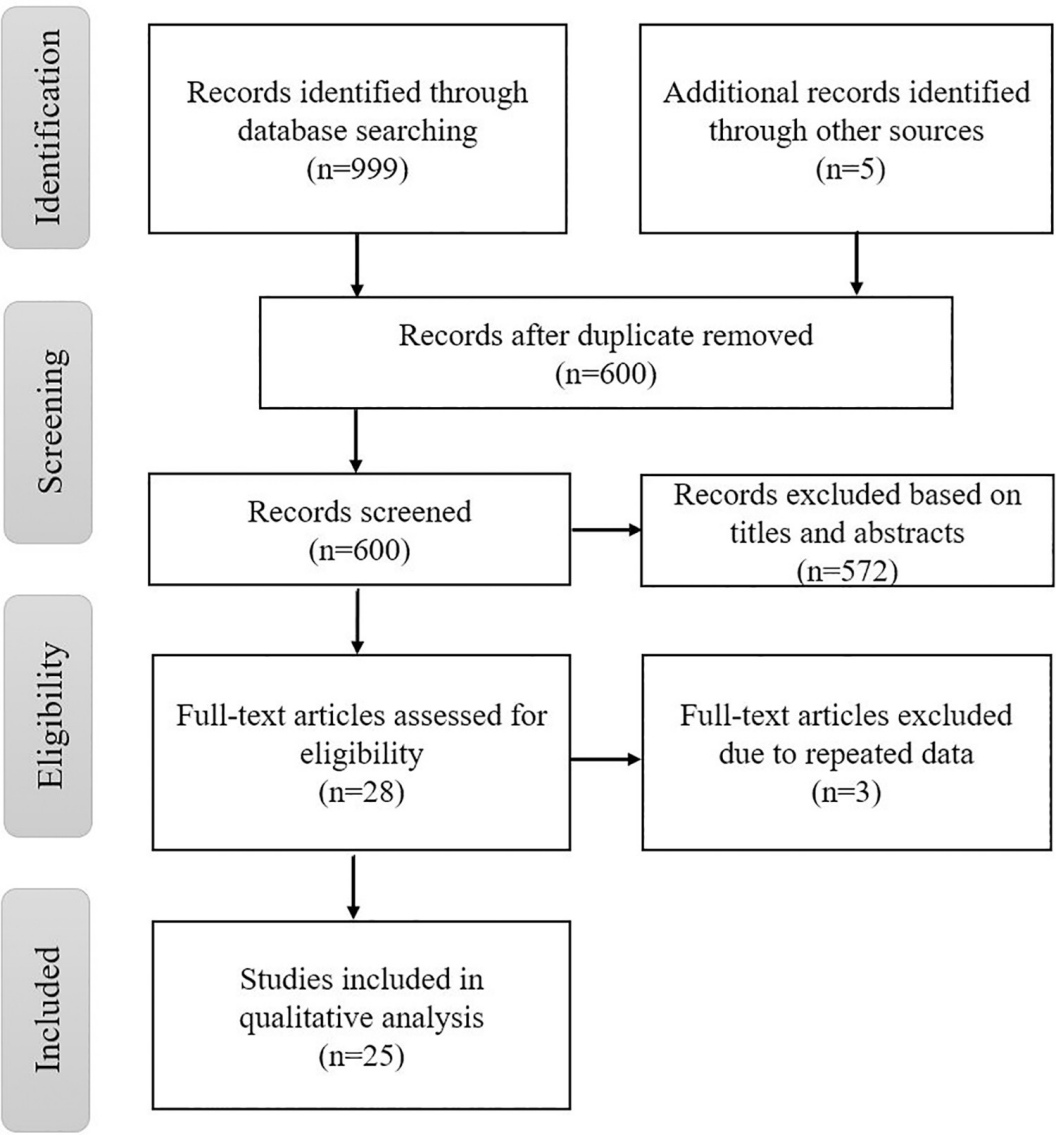
PRISMA flow diagram of literature search for the systematic review. The PRISMA flow diagram depicts the flow of information throughout the different phases of this systematic review, including the number of records identified, included, and excluded and the reasons for the exclusions.

The electronic database search revealed 999 records: 246 studies from PubMed; 291 from ScienceDirect; 446 from Google Scholar; and 16 from IEEE Xplore. After removing 404 duplicates, 572 studies were excluded based on the exclusion criteria, leaving 28 potentially relevant studies. Most of the excluded studies did not report ligament loading data despite the inclusion of ligaments in the models. Others were based on finite element modelling or other non-musculoskeletal modelling approaches. Three of these studies were also excluded due to repeated data. Of the 1004 original records only 25 meet the inclusion criteria and were included in this review. The study selection process is reported in Fig 1. Quality assessment and data extraction results are reported below. Details can be found in Tables 2–4, respectively.

**Table 2:** Quality assessment results of included publications database (25 publications).

Study	Year	Question number																
		1	2	3	4	5	6	7	8	9	10	11	12	13	14	15	16	%
Frigo et al. [14]	2021	2	2	2	2	2	2	0	2	2	1	2	2	2	2	2	2	90.62
Kim et al. [15]	2021	2	2	2	2	2	1	0	1	2	2	2	1	2	2	2	2	84.37
Moon et al. [16]	2021	2	2	2	2	1	1	0	2	2	0	2	2	2	2	2	2	81.25
Sikidar et al. [17]	2021	2	2	2	2	2	1	0	2	2	0	2	2	2	2	2	2	84.37
Vignos et al. [18]	2020	2	2	2	2	2	2	2	2	2	1	2	2	2	2	2	2	96.87
Tanaka et al. [19]	2020	2	1	1	2	1	1	2	2	2	2	2	2	2	2	2	2	87.50
Nasseri et al. [20]	2020	2	2	2	1	1	1	0	2	2	2	2	2	2	2	2	2	84.37
Charles at al. [21]	2020	2	2	2	2	2	2	2	2	2	2	2	2	2	2	2	2	100
Smale et al. [22]	2019	2	2	2	2	2	1	1	2	2	2	2	2	2	2	2	2	96.87
Blache et al. [23]	2019	2	2	2	1	2	2	2	2	2	2	2	2	2	2	2	2	96.87
Barzan et al. [24]	2019	2	2	2	2	2	2	0	2	2	2	2	2	2	2	2	2	93.75
Marieswaran et al. [25]	2018	2	2	2	1	2	1	0	2	2	1	2	2	2	2	2	2	84.37
Hu et al. [26]	2018	2	2	2	2	2	1	0	2	2	2	2	2	1	2	2	2	87.50
Moon et al. [27]	2018	2	2	2	1	2	1	0	2	2	0	2	2	2	2	2	2	81.25
Kang et al. [28]	2017	2	2	2	2	2	2	2	2	2	2	2	2	2	2	2	2	100
Schmitz et al. [29]	2016	2	2	2	2	2	2	2	2	2	2	2	2	2	2	2	2	100
Kia et al. [30]	2016	2	2	2	2	2	1	1	2	2	2	2	2	2	2	2	2	93.75
Bersini et al. [31]	2016	2	2	2	2	2	1	0	2	2	2	2	2	2	2	2	2	90.62
Xu et al. [32]	2015	2	2	2	2	2	2	2	2	2	2	2	2	2	2	2	2	100
Ozada et al. [33]	2015	2	2	2	1	1	1	1	2	2	2	2	2	2	2	2	2	87.5
Kar et al. [34]	2012	2	2	2	2	2	1	1	2	2	2	2	2	2	2	2	2	93.75
Shao et al. [35]	2011	2	2	2	2	1	2	2	2	2	1	2	2	2	2	2	2	93.75
Shelburne et al. [36]	2004	2	2	2	2	2	2	2	2	2	2	2	2	2	2	2	2	100
Shelburne et al. [37]	2002	2	2	2	2	1	2	0	2	2	1	2	2	2	2	2	2	87.5
Shelburne et al. [38]	1997	2	2	2	2	1	2	0	2	2	0	2	2	2	2	2	2	84.37

Items were scored from 0 to 2. Questions related to the description or justification of (1) Objectives; (2–4) Study Design; (5–7) Modelling Technique; (8) Movement Tasks; (9) Simulation; (10) Validation; (11) Statistics; (12) Results; (13–14) Key Findings; (15) Limitations; (16) Conclusion.

**Table 3:** Musculoskeletal knee joint modelling characteristics of included publications (in descending chronological order).

**Study & Year**	**Modelling Software**	**Knee Type**	**Model Type**	**Source of Geometry**	**Cartilage**	**Contact**	**Menisci**	**DoFs**	**Subjects**	**Type of Activity**	**Collected Data**	**Simulation**
Frigo et al. [14], (2021)	SimWise-4D platform	Intact	Lower-Body	MRI	Yes	No	No	6 TF	5 participants with intact knee	Walking	Motion capture data, GRF	IK, ID, FD, SO
Kim et al. [15], (2021)	OpenSim	Intact	Whole-body	Scaled generic model	No	No	No	5 TF	10 participants with intact knee	Single-leg landing	Motion capture data, GRF, EMG	IK, ID, RRA, PK, CMS
Moon et al. [16], (2021)	OpenSim	Intact	Whole-body	Scaled generic model	No	No	No	3 TF	15 participants with intact knee	Walking, Running, Direction diversion manoeuvre	Motion capture data, GRF	IK, ID, RRA, CMC, FD
Sikidar et al. [17], (2021)	OpenSim	Intact	Lower-Body	Scaled generic model	No	Femoral Cartilages	Yes	6 TF	4 participants with intact knee	Walking, Plant-and-cut	Motion capture data	IK
Vignos et al. [18], (2020)	SIMM	ACLR	Lower-Body	MRI	Yes	Femoral / Tibial Cartilages	Yes	5 TF	18 patients with ACLR knee	Walking	Dynamic MRI, Motion capture data, GRF	IK, ID, SO
Tanaka et al. [19], (2020)	KneeSIM	Intact	One leg	NR	No	No	No	NR	NR	Walking, Stepping, Squatting	Motion capture data, GRF, Fluoro	IK
Nasseri et al. [20], (2020)	OpenSim	Intact	Whole-body	Scaled generic model	No	NR	No	6 TF	13 participants with intact knee	Drop Landing	Motion capture data, GRF, EMG	IK, ID, MA
Charles at al. [21], (2020)	OpenSim	Intact	One leg	CT/ Scaled generic model	No	No	No	6 TF	10 participants with intact knee	Treadmill walking	MRI, CT, Motion Capture Data, GRF, biplane radiography	IK, ID, RRA, SO
Smale et al. [22], (2019)	OpenSim	Intact	Whole-body	MRI	No	Four Spherical Surfaces	No	6 TF	11 patients with ACLD knee	Side Cut	MRI, Motion capture data, GRF, Video Fluoro	IK
Blache et al. [23], (2019)	OpenSim	LER	One leg	CT	No	No	No	6 TF	One participant with intact knee	Squatting	CT, X-ray	FD
Barzan et al. [24], (2019)	MultiBody	Intact	One leg	MRI	No	Four Spherical Surfaces	No	5 TF, 6 PF	Eight participants with intact knee	Passive knee flexion	MRI	IK, OPT
Marieswaran et al. [25], (2018)	OpenSim	Intact	Lower-Body	MRI	Femoral only	Femoral Cartilages	Yes	6 TF, 1 PF	One intact cadaveric knee	Passive knee flexion, internal rotation, and adduction	MRI, Passive knee movement	FD
**Study & Year**	**Modelling Software**	**Knee Type**	**Model Type**	**Source of Geometry**	**Cartilage**	**Contact**	**Menisci**	**DoFs**	**Subjects**	**Motion**	**Collected Data**	**Simulation**
Hu et al. [26], (2018)	AnyBody	Intact	Whole-body	MRI	Yes	Femoral / Tibial Cartilages	Yes	6 TF, 5 PF	One intact cadaveric knee	Walking	MRI, Motion capture data, GRF	IK, ID
Moon et al. [27], (2018)	OpenSim	Intact	Whole-body	Scaled generic model	No	No	No	3 TF	19 participants with intact knee	Drop jump	Motion capture data, GRF	IK, RRA, ID, CMC, FD
Kang et al. [28], (2017)	AnyBody	Intact, PCLD	Whole-body	Scaled generic model	Yes	Femoral / Tibial Cartilages	No	6 TF, 6 PF	One participant with intact knee	Walking, Squatting	MRI, CT, Motion capture data, GRF, EMG	ID, MC
Schmitz et al. [29], (2016)	OpenSim	Intact	One leg	MRI	Femoral only	Femoral Cartilage/ Tibial Plane	No	6 TF, 1 PF	One participant with intact knee	Passive knee flexion, internal rotation, and adduction	Passive knee movement	FD, SO, CMC
Kia et al. [30], (2016)	ADAMS	Intact	One leg	CT	Yes	Femoral / Tibial Cartilages	Yes	6 TF	One intact cadaveric knee	Passive knee flexion	CT, Digitisers	OPT, MA
Bersini et al. [31], (2016)	Working Model 3D	Intact	One leg	MRI	No	Femoral / Tibial Cartilages	No	5 TF	One participant with intact knee (MRI only)	Passive squatting, knee flexion, hyperextension, adduction, free hung, AP drawer test	MRI	NR
Xu et al. [32], (2015)	OpenSim	Intact	Lower-Body	Scaled generic model	No	No	No	6 TF	SIMM Generic Model	Passive knee flexion, internal rotation, and adduction	Passive knee movement	FD
Ozada et al. [33], (2015)	MSK Joint Modeler	Intact	One leg	3D Laser Scanning	Yes	Femoral / Tibial Cartilages	No	6 TF	Plastic sawbones	Passive knee flexion	3D laser scanning	MA
Kar et al. [34], (2012)	OpenSim	Intact	Whole-body	Scaled generic model	No	TF Contact Forces	No	6 TF	OpenSim generic model	Stop Jumping-Height	Motion capture data, GRF, EMG	IK, FD
Shao et al. [35], (2011)	OpenSim	Intact, ACLD	Lower-Body	Scaled generic model	No	TF Contact Forces	No	3 TF, PF is NR	One participant with intact knee/ 1 patient with ACLD knee	Walking, Running	Motion capture data, GRF, EMG, Dynamometer	IK, ID, FD
Shelburne et al. [36], (2004)	SIMM	Intact, ACLD	One leg	CT	No	TF Contact Forces	No	6 TF, 1 PF	Five participants with intact knee	Walking	Motion capture data, GRF, EMG	ID, FD
Shelburne et al. [37], (2002)	SIMM	Intact	Lower-Body	Scaled generic model	No	TF Contact Forces	No	3 TF	Five participants with intact knee	Squatting	MRI, Motion capture data, GRF, EMG	OPT, MA
Shelburne et al. [38], (1997)	SIMM	Intact	Lower-Body	Literature	No	TF Contact Forces	No	3 TF	Five participants with intact knee	Passive knee flexion, AP drawer test	Dynamometer	NR

**Abbreviations**: ACLD- Anterior Cruciate Ligament Deficient, ACLR- Anterior Cruciate Ligament Reconstruction, AP- Anterior Posterior, CT- Computed Tomography, CMC- Computed Muscle Control, FD- Forward Dynamics, GRF- Ground Reaction Forces, ID- Inverse Dynamics, IK- Inverse Kinematics, LER- Lateral Extraarticular Reconstruction, MA- Muscle Analysis, MC- Monte Carlo, MRI- Magnetic Resonance Imaging, MSK- Musculoskeletal, NR- Not Reported, OPT- Optimisation, PCLD- Posterior Cruciate Ligament Deficient, PF- Patella Femoral, PK- Point Kinematics, RRA- Residual Reduction Algorithm, SO- Static Optimisation, TF- Tibio Femoral.

**Table 4:** Ligament model characteristics of included publications (in descending chronological order).

Study & Year	Ligament Bundles	Attachment Points Identification	Material Property (Stiffness, Reference, or Slack Length)	Ligament Pathway	Predicted Ligament Mechanics Parameter
Frigo et al. [14], (2021)	ACL (AM, PL), PCL (AM, PL), aMCL, iMCL, pMCL, aDMCL, pDMCL, LCL, CAPa, CAPl, CAPm	Literature	Literature	Straight Line	Force
Kim et al. [15], (2021)	ACL (AM, PL), PCL (AM, PL)	From Generic Model	Literature	NR	Force
Moon et al. [16], (2021)	ACL (AM, PL), PCL (AM, PL)	Literature	Literature	NR	Force
Sikidar et al. [17], (2021)	ACL	Literature	Literature	Curvilinear Path	Force/Strain
Vignos et al. [18], (2020)	ACL graft, MCL, LCL, PFL, CAP	MRI	MRI	Straight Line	Force
Tanaka et al. [19], (2020)	ACL (AM, PL), PCL (AM, PL), aMCL, pMCL, LCL	Literature	Literature	NR	Force
Nasseri et al. [20], (2020)	ACL, PCL, MCL, LCL	From Generic model	NR	Straight Line	Force
Charles at al. [21], (2020)	ACL (AM, PL), PCL (AM, PL)	MRI	Literature	Straight Line	Force
Smale et al. [22], (2019)	ACL, PCL, MCL, LCL	MRI	NR	Curvilinear Path	Length
Blache et al. [23], (2019)	ALL graft, NR about other ligaments	From Generic model	Literature	Curvilinear Path	Force
Barzan et al. [24], (2019)	ACL, PCL, MCL, LCL	MRI	MRI (Length only)	Curvilinear Path	Strain
Marieswaran et al. [25], (2018)	ACL, PCL, MCL, LCL, CAP, MFL, PFL, TL	Literature	Literature	Curvilinear Path	Force/Strain
Hu et al. [26], (2018)	ACL, PCL, aMCL, iMCL, pMCL, LCL, ALL, CAP, OPL	From Generic model	Literature	NR	Force
Moon et al. [27], (2018)	ACL	Literature	Literature	NR	Force
Kang et al. [28], (2017)	ACL, PCL, MCL, LCL, mPFL, lPFL, CAP, OPL	MRI	Literature	Curvilinear Path	Force
Schmitz et al. [29], (2016)	ACL, PCL, aMCL, iMCL, pMCL, aDMCL, pDMCL, LCL, PFL, CAPa, CAPl, CAPo, CAPm	Literature	Literature	Curvilinear Path	Force/Strain
Kia et al. [30], (2016)	ACL (AM, PL), PCL (AL, PM), MCL, LCL, ALL, PFL, CAPl, CAPm, OPL	CT, Dissection, Literature	CT, Dissection, Literature	Straight Line	Force
Bersini et al. [31], (2016)	ACL (AM, PL), PCL (AL, PM), aMCL, iMCL, pDMCL, LCL	MRI	Literature	NR	Force
Xu et al. [32], (2015)	ACL (AM, PL), PCL (AL, PM), aMCL, iMCL, pMCL, aDMCL, pDMCL, LCL	Literature	Literature	Straight Line	Strain
Ozada et al. [33], (2015)	ACL, PCL, MCL, LCL	From Generic model	From Generic Model	Curvilinear Path	Length
Kar et al. [34], (2012)	ACL, Bicep Femoris Tendons, Gastrocnemius Tendons	Literature	Literature	Straight Line	Force/Strain
Shao et al. [35], (2011)	ACL (AM, PL), PCL (AL, PM), aMCL, iMCL, pMCL, aDMCL, pDMCL, LCL, ALL, CAP	Literature	Literature	Straight Line	Force
Shelburne et al. [36], (2004)	ACL (AM, PL), PCL (AL, PM), aMCL, iMCL, pMCL, aDMCL, pDMCL, LCL, ALL, CAPl, CAPm	Literature	Literature	Curvilinear Path	Force
Shelburne et al. [37], (2002)	ACL (AM, PL), PCL (AL, PM), aMCL, iMCL, pMCL, aDMCL, pDMCL, LCL, CAP	Literature	Literature	Straight Line	Force
Shelburne et al. [38], (1997)	ACL (AM, PL), PCL (AL, PM), aMCL, iMCL, pMCL, aDMCL, pDMCL, LCL, CAP	Literature	Literature	Straight Line	Force

Abbreviations: ACL-Anterior Cruciate Ligament, aDMCL- anterior bundle of Deep Medial Collateral Ligament, AM- Anteromedial, aMCL- anterior bundle of Medial Collateral Ligament, ALL- Antero Lateral Ligament, CAP- posterior Capsule, CAPl- lateral bundle of posterior Capsule, CAPm- medial bundle of posterior Capsule, CAPa- arcuate popliteal of posterior Capsule, CAPo- oblique Popliteal bundle of posterior capsule, iMCL- central bundle of Medial Collateral Ligament, LCL- Lateral Collateral Ligament, lPFL- lateral Patello-Femoral Ligaments, MFL-Menisco-Femoral Ligament, MRI-Magnetic Resonance Imaging, mPFL- medial Patello-Femoral Ligaments, NR-Not Reported, OPL-Oblique Popliteal Ligament, PCL- Posterior Cruciate Ligament, pDMCL- posterior bundle of Deep Medial Collateral Ligament, PFL- Popliteofibular Ligament, PL- Posterolateral, pMCL- posterior bundle of Medial Collateral Ligament, TL- Transverse Ligament.

### Quality assessment

The quality of the reviewed studies is summarised in Table 2. Each article’s overall score is calculated by the sum of the rated questions divided by the sum of relevant questions. The selected studies demonstrated high quality in the areas of objectives, study design, modelling technique, movement tasks, simulation, validation, statistics, results, key findings, limitations, and conclusion.

According to the quality assessment results presented in Table 2, several articles had limited modelling technique descriptions [15, 16, 19, 20, 27, 33] and validation methods [16, 18, 25, 27, 35, 37, 38]. In 12 of the selected studies [14–17, 20, 24–27, 31, 37, 38], the sensitivity of the model outputs on ligament model parameters has not been assessed. In one study [26], findings were not fully supported by the literature. Overall, the selected articles demonstrated high content quality, with scores ranging between 81.25% and 100%.

### Musculoskeletal model definition

**Fig 2:**
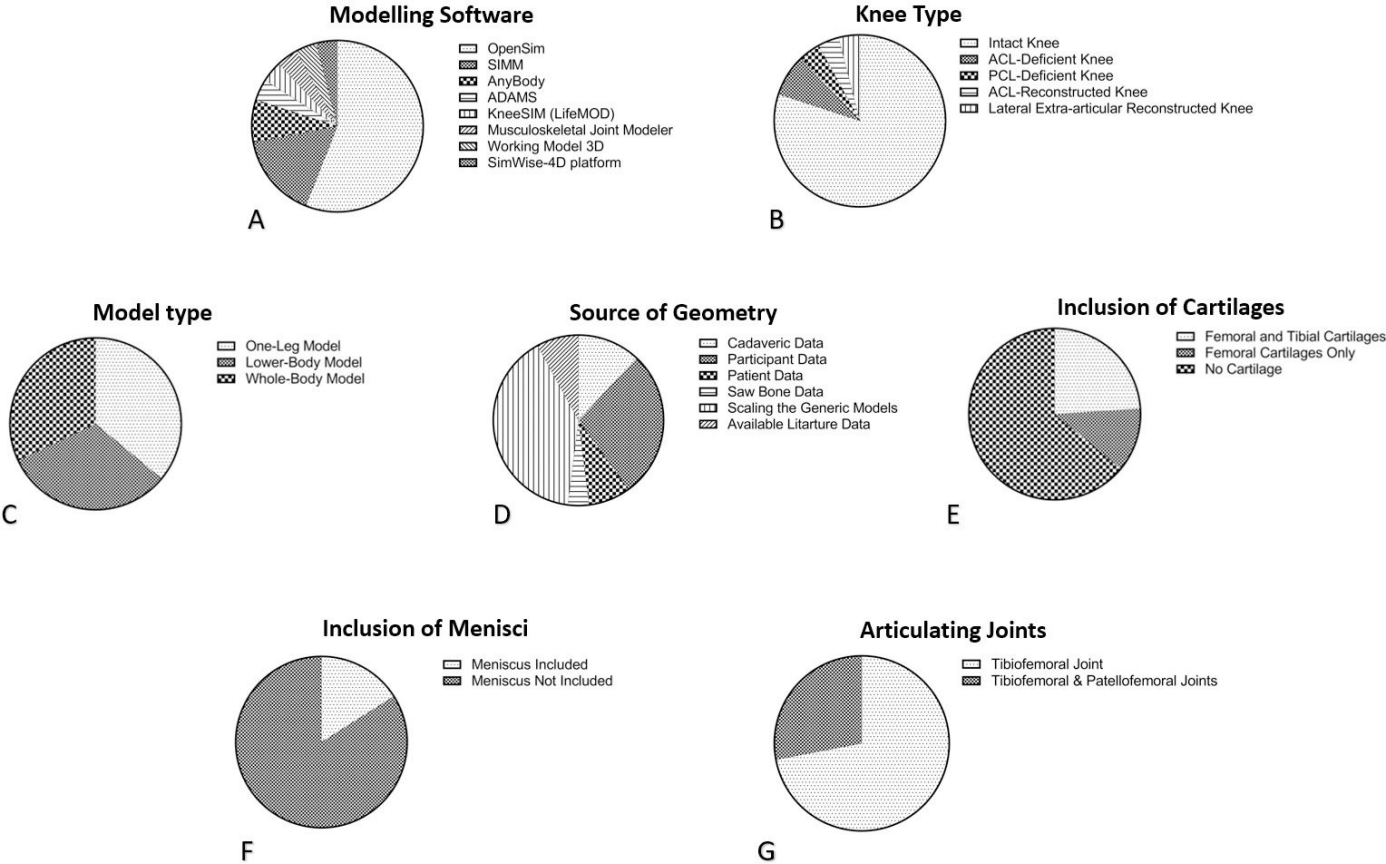
Distribution of studies by model characteristics. Pie charts representing the distributions of studies according to various modelling parameters: modelling software (A), knee type (B), model type (C), source of geometry (D), inclusion of cartilages (E), inclusion of menisci (F), and articulating joints forming the knee (G).

Details of the extracted musculoskeletal model definitions are summarised in three sections: geometry, degrees of freedom/joint boundary conditions, and ligament modelling, with detailed information presented in Tables 3 and 4, and Fig 2.

A range of musculoskeletal modelling software packages has been used across the selected studies (Fig 2A and Table 3). 14 studies [15–17, 20–25, 27, 29, 32, 34, 35] used OpenSim [39], four studies [18, 36–38] used SIMM [40], two studies [26, 28] used AnyBody Modelling System (AnyBody Technology, Aalborg, Denmark), one study [30] used ADAMS [41], one study [14] used SimWise-4D platform (Design Simulation Technologies, DST, Canton, MI, USA), one study [19] used KneeSIM (LifeModeler Inc., San Clemente, CA), one study [33] used Musculoskeletal Joint Modeler [42], and one study [31] used Working Model 3D (Working Model 3D, MSC, Software Corp., Santa Ana, CA, USA).

#### Geometry

Out of the 25 studies included, 20 studies were designed to simulate the intact knee joint [14–17, 19–22, 24–27, 29–34, 37, 38], two studies modelled both intact and ACL-deficient knee [35, 36], one study modelled the intact and PCL-deficient knees [28], one study modelled the ACL-reconstructed knee [18], and one study modelled the lateral extra-articular reconstructed knee [23] (Fig 2B and Table 3). Nine studies modelled one leg [19, 21, 23, 24, 29–31, 33, 36], eight studies developed the whole-body models [15, 16, 20, 22, 26–28, 34] and eight studies modelled the lower-body [14, 17, 18, 25, 32, 35, 37, 38] (Fig 2C and Table 3).

Knee models were generated for 53 intact knees, 18 ACL-reconstructed knees, and 12 ACL-deficient knees. These models included geometry from 127 participants/patients’ knees and three cadaveric knees. Intact knee geometries were extracted fully or partially from segmented medical imaging (CT or MRI) of cadaveric specimens [25, 26, 30], participants [14, 21, 23, 24, 29, 31, 36], patients [18, 22], saw bone [33] or by scaling the generic reference models based on anthropometric data of study participants [15–17, 20, 21, 27, 28, 32, 34, 35, 37] (Fig 2D and Table 3). In the rest of the studies, the geometry was taken from available data in the literature [19, 38].

Six studies included the femoral and tibial cartilages [14, 18, 26, 28, 30, 33] and three studies had the femoral cartilage only [17, 25, 29] (Fig 2E and Table 3). The remainder of the studies did not consider cartilage geometry. Only four studies modelled the menisci [18, 25, 26, 30] (Fig 2F and Table 3), and seven studies modelled the patella [24–26, 28, 29, 35, 36].

#### Joint boundary conditions (DoFs and type of activity)

In eight studies, the knee joint model included both the tibiofemoral and patellofemoral joints [24–26, 28, 29, 35–37] (Fig 2G and Table 3). The remainder modelled only the tibiofemoral joint. The number of degree of freedoms (DoFs) available in the knee joint was primarily related to each study’s generic musculoskeletal model and ranged between 3-DoFs, where only the flexion/extension angle, anterior-posterior and proximal-distal translations of the tibiofemoral joint were considered [37, 38] up to a 12-DoF model, which considered flexion-extension, abduction-adduction and tibial internal-external rotations and anterior-posterior, proximal-distal, and medial-lateral translations for both the tibiofemoral and patellofemoral joints [28].

Models were used to simulate various active motions including walking [14, 17–19, 21, 26, 28, 35, 36], running [16, 35], squatting [19, 23, 28, 37], stepping [19], side cutting[22], stop jumping-height [34], single-leg landing [15], drop-landing [20, 27], and passive motions including passive knee flexion [24, 25, 29–33, 38], internal rotation [25, 29, 32], hyper-extension [31], adduction [25, 29, 31, 32], passive squatting [31], drawer test [31, 38], and free hung [31].

#### Ligament modelling

The different ligament modelling techniques are detailed below and summarised in Table 4. Among the 25 selected studies, ligament bundle attachment footprints were identified directly from cadaveric knees for one study [30] and virtually on the 3D bone models generated from MRI data in six studies [18, 21, 22, 24, 28, 31]. Other studies used literature to define the attachment points [14–17, 19, 20, 23, 25–27, 29, 32–38]. Generally, ligaments with small attachment footprints were modelled as a single fibre connecting the centroids of ligament insertion sites [18, 20, 22, 24–30, 33, 34, 43], and ligaments with larger footprints, with multiple fibres [14–16, 19, 21, 23, 31, 32, 35–38].

The fibre path was modelled either as straight lines connecting insertion points [14, 18, 20, 21, 30, 32, 34, 35, 37, 38] or as curved lines passing over curved wrapping objects [22–25, 28, 29, 33, 43] (Table 4). Wrapping objects are included in the knee joint model to prevent the ligament’s penetration into the bone and cartilage geometries.

Ligament reference length is the ligament length at a given reference position and is used to normalise the length change measurement. The definition of the reference length varied between studies, nine studies using the length at full knee extension [23–25, 29–33, 38], ten studies using the length at heel strike during walking [14, 16–19, 21, 25, 26, 28, 35] and three studies using the length at initial heel contact at landing after jumping down from a height [15, 27, 34]. These reference lengths were either determined using MRI scans [18, 21, 22, 24, 28, 31], extracted from a scaled generic model [15, 20, 23, 26, 33] or defined based on literature data [14, 16, 17, 19, 25, 27, 29, 32, 34, 35, 37, 38] (Table 4).

Ligament stiffness values were generally retrieved from previous literature studies [14–17, 19, 21, 23, 25–32, 34–38]. However, Vignos et al. [18] computed the stiffness for individual ligaments as the product of the ligament cross-sectional areas, as measured from MRI scans, and an assumed elastic modulus of 125 MPa. Otherwise, the generic model’s predefined stiffness values were used to define the ligament properties [33] or no information was reported about the stiffness values used [20, 22, 24].

### Sensitivity analysis

Several different methods have been reported to assess ligament loading behaviour’s sensitivities to the modelling parameters using computer simulation models [18, 19, 21–23, 28, 29, 32, 35] (Table 5).

**Table 5:** Comparison of different validation approaches and sensitivity analysis between included publications (in descending chronological order).

Study	Year	Validation							Sensitivity Analysis			
		Method		Validated Parameter					Assessed Parameter			
		In vivo	In vitro	Kinematics	Contact force	Muscle force	Ligament force	Ligament strain\length	Active kinematics	Passive kinematics	Ligament force	Ligament strain\length
**Frigo et al. [14]**	2021	✓		✓	✓							
**Kim et al. [15]**	2021	✓				✓						
**Moon et al. [16]**	2021											
**Sikidar et al. [17]**	2021		✓				✓					
**Vignos et al. [18]**	2020	✓	✓	✓							✓	
**Tanaka et al. [19]**	2020	✓		✓					✓		✓	✓
**Nasseri et al. [20]**	2020	✓	✓			✓	✓					
**Charles at al. [21]**	2020	✓		✓	✓						✓	
**Smale et al. [22]**	2019		✓	✓					✓			✓
**Blache et al. [23]**	2019	✓		✓					✓			✓
**Barzan et al. [24]**	2019	✓		✓								
**Marieswaran et al. [25]**	2018											
**Hu et al. [26]**	2018	✓		✓	✓	✓						
**Moon et al. [27]**	2018											
**Kang et al. [28]**	2017	✓		✓	✓	✓				✓		✓
**Schmitz et al. [29]**	2016		✓	✓	✓			✓		✓		✓
**Kia et al. [30]**	2016		✓	✓			✓					
**Bersini et al. [31]**	2016	✓			✓	✓						
**Xu et al. [32]**	2015		✓					✓				✓
**Ozada et al. [33]**	2015		✓	✓				✓				
**Kar et al. [34]**	2012	✓		✓		✓						
**Shao et al. [35]**	2011	✓	✓	✓		✓	✓				✓	
**Shelburne et al. [36]**	2004	✓		✓		✓						
**Shelburne et al. [37]**	2002	✓		✓		✓						
**Shelburne et al. [38]**	1997		✓	✓								

Three studies focused on the sensitivity of the *active* knee kinematics to varying ligament lengths [19, 22, 23]. For instance, Tanaka et al. [19] found that active knee kinematic are sensitive to ACL slack length, and Blache et al. [23] found a similar sensitivity to LER attachment site, with a postero-proximal femoral LER attachment site leading to active kinematics closer to an intact knee. In another study, Smale et al. [22] found that the model’s knee kinematics and ligament length were very sensitive to the joint geometry and contact surfaces during highly dynamic tasks.

*Passive* knee kinematics have been shown to be sensitive to ligament parameters [28, 29]. Schmitz et al. [29] found that passive knee kinematics are sensitive to the slack length and stiffness of the ACL, PCL, and MCL. Kang et al. [28] showed that tibiofemoral translations and internal tibial rotation were sensitive to the stiffness and strain values at full extension within the PFL, LCL, and PT ligaments.

The sensitivity of ligament model parameters on forces experienced by ACL [18, 21, 35] and MCL [19, 35] have also been assessed. Vignos et al. [18] found that forces in the ACL during walking were sensitive to vertical graft angle, tibial translation, and initial tension in the graft. Tanaka et al. [19] reported that the MCL tension during deep knee bend, gait, and stair descent activities was sensitive to ACL length change. Charles et al. [21] demonstrated the sensitivity of ACL force predictions to subject-specific anatomy, specifically musculoskeletal joint geometry and ligament resting lengths. They showed that during walking, ACL forces were highly sensitive to the ligament resting length with ±10% variations resulting in force differences of up to 84%. Shao et al. [35] revealed the sensitivity of ACL and MCL forces during the stance phase to variations in different anterior tibial translations.

One study assessed the effect of variation in the slack length and ligament strain on the ligament strain errors [32]. It was found that in an OpenSim generic ligament model, ligament strain error was highly sensitive to variations in ligament strain and slack length.

### Validation approaches

An essential stage of modelling is validation, which compares the performance of the models against experimental measurements. Two different types of measurements were used for validation: *in vivo* experimental data and *in vitro* experimental data (Table 5). Only three reviewed papers did not include any form of validation [16, 25, 27].

Among the selected studies in this systematic review, 15 studies used *in vivo* experimental datasets to validate their model predictions [14, 15, 18–21, 23, 24, 26, 28, 31, 34–37]. Common *in vivo* datasets used to validate simulation data includes kinematics derived from the fluoroscopic analysis [19], CT/MR/X-ray image analysis [21, 23, 24, 28]; motion capture analysis [14, 20]; EMG results [15, 31]; or a combination of these techniques [18, 26, 31, 34–37]. A combination of active [14, 15, 17–19, 21, 23, 26, 31, 34–37] and passive [18, 20, 24, 28] activities were used to generate these data sets.

*In vitro* validation methods compare model performance against kinematic and kinetic data measured in cadaveric testing. For example, Smale et al. [22] used a modelling method previously validated against cadaveric passive flexion data [44] and Kia et al. [30] collected ligament forces and experimental kinematics during the passive flexion of a cadaveric knee to validate their model. Among the nine studies which validated their models against in vitro cadaveric experiments [18, 20, 22, 29, 30, 32, 33, 35, 38], some used knee kinematics [18, 22, 38]; others used ligament strain/force data [20, 32]; some used a combination of these [29, 30, 33, 35].

### Model predictions

All models in this systematic review report ligament mechanics in one form or another (Table 4). Depending on the research question, either the ligaments’ force, strain, or length has been reported.

#### Predicted forces

**Fig 3:**
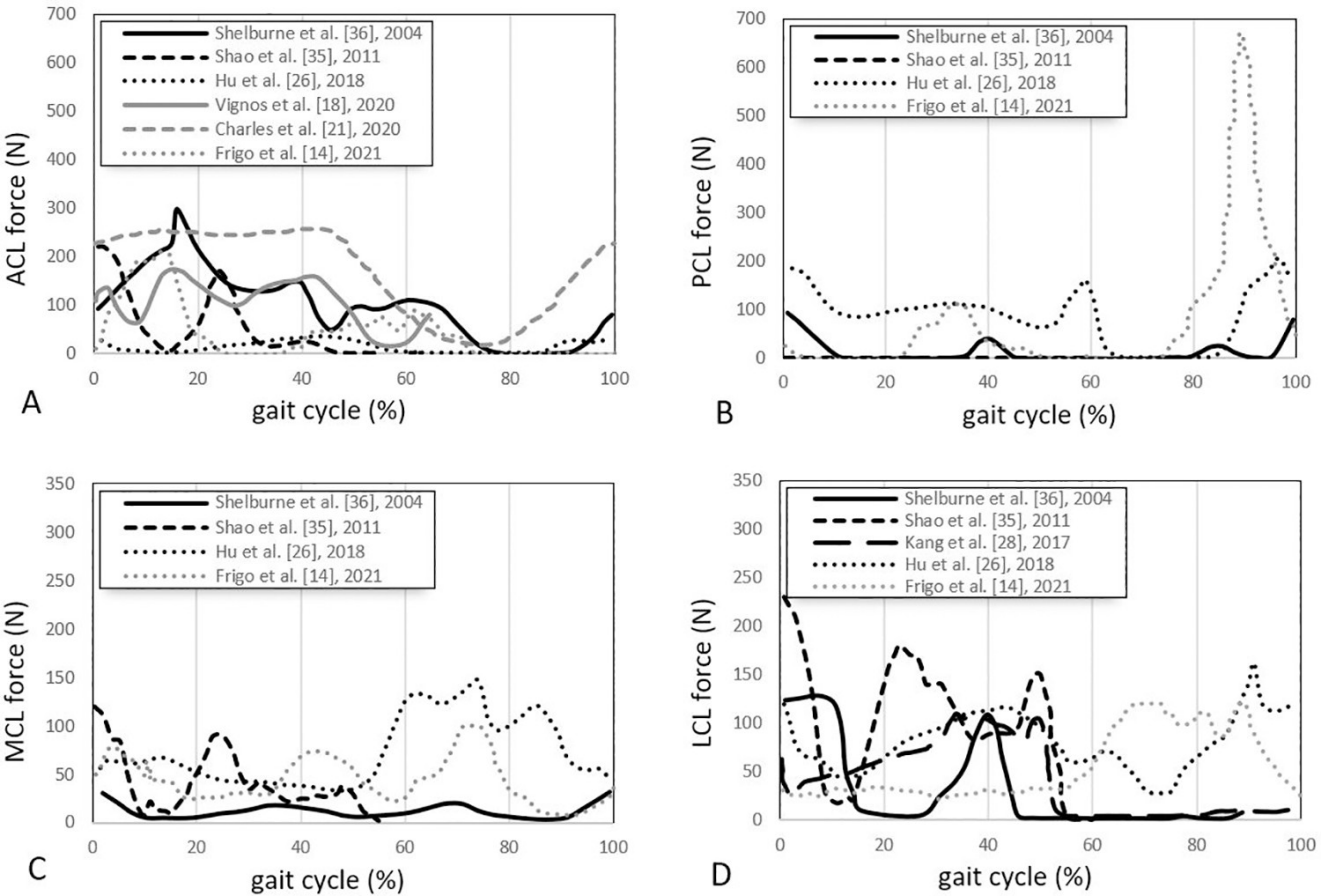
Reported ACL, PCL, MCL, and LCL forces vs percentage of the gait cycle during walking in different studies [14, 18, 21, 26, 28, 35, 36]. Curves represent the trends of ligament forces (in Newton) experienced by ACL (A), PCL (B), MCL (C), and LCL (D) during the stance phase (0–60%) and the swing phase (60–100%) of the gait cycle.

**Fig 4:**
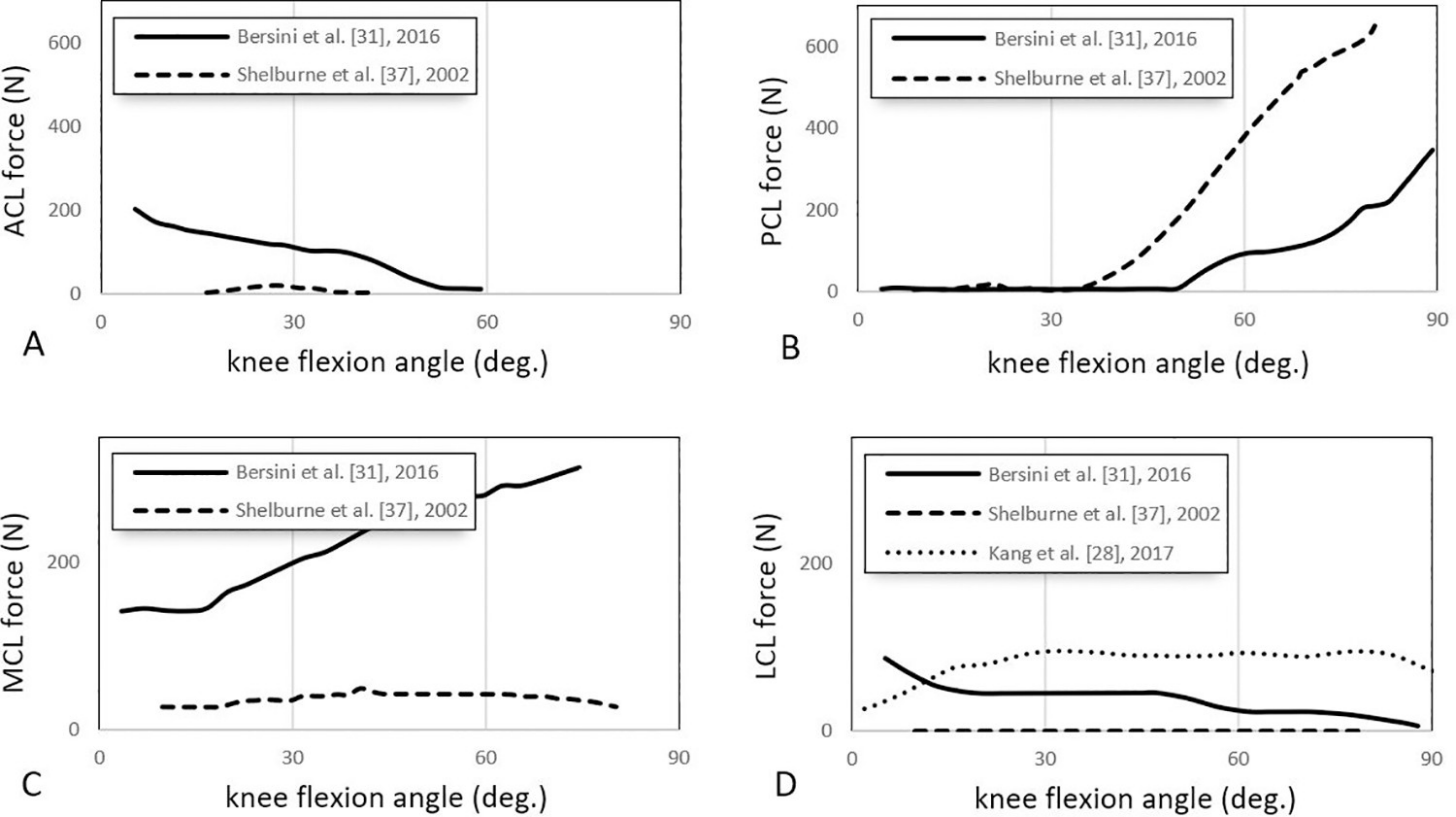
Reported ACL, PCL, MCL and LCL forces vs knee flexion angle during passive [31] and active [28, 37] squatting in three different studies. Curves represent the trends of ligament forces (in Newton) experienced by ACL (A), PCL (B), MCL (C), and LCL (D) in 0° to 90° of knee flexion along with the squat movement. To highlight the trend more clearly, the MCL and LCL forces are presented in the 0-300N range.

**Fig 5:**
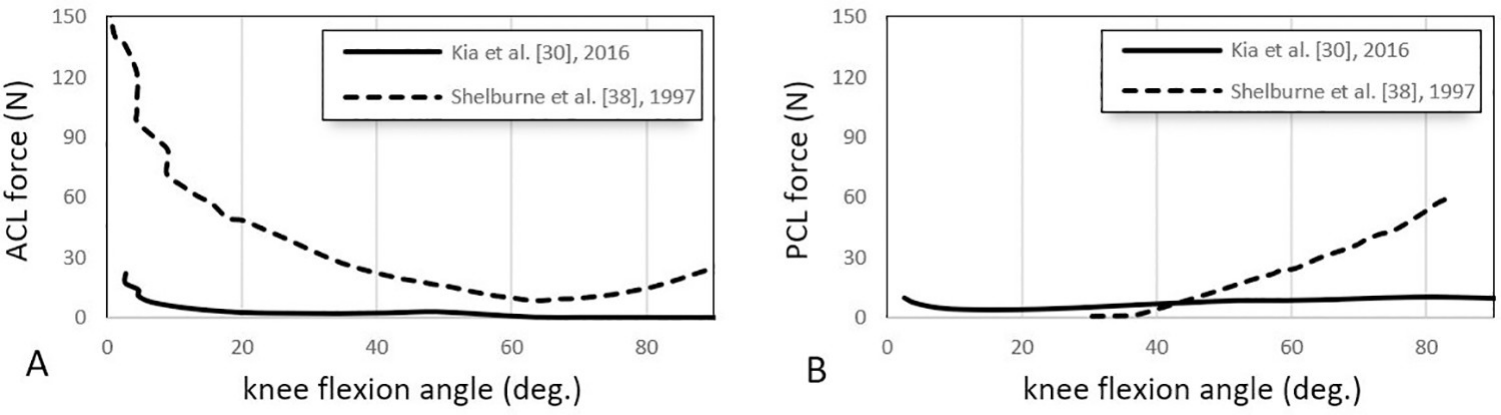
Reported ACL and PCL forces vs knee flexion angle during passive knee flexion in two different studies [30, 38]. Curves represent the trends of ligament forces (in Newton) experienced by ACL (A) and PCL (B) in 0° to 90° of knee flexion along with the passive flexion movement.

Fourteen studies reported ligament forces [18–20, 23, 25, 26, 28–31, 34–38], incorporating passive and active activities. Typically, walking [14, 17, 18, 21, 26, 28, 35, 36] and squatting [28, 31, 37] are used for simulating active movements, and passive knee flexion [30, 38] and the anterior drawer test [31] are used as passive movements. Figs 3 and 4 show the comparative ligament force values across the different studies for *active* movements, including walking and squatting. Fig 5 reports the ligament for *passive* knee flexion.

*Walking (active)*. Six studies [14, 18, 21, 26, 35, 36] calculated the ACL forces of healthy knees using their models under simulated dynamic walking conditions (Fig 3A). Five showed the ACL tensions ranging between 50-300N [14, 18, 21, 35, 36]. They generally found the ACL active during the stance phase. All three studies showed peaks in the ACL force in the midstance phase (15%–45% of the gait cycle). However, there was considerable variation in the distribution of those peaks. In contrast, Hu et al. [26] reported low tensions (less than 50N) in the ACL throughout the whole gait cycle. Three studies [14, 26, 36] (Fig 3B) reported two force peaks in the PCL associated with the ipsilateral heel strike and toe-off, with a comparatively high peak of PCL force (approximately 700N) reported by Frigo et al. [14] at toe-off. In contrast, Hu et al. [26] suggest that the PCL is loaded throughout the stance phase, while the other three studies report comparatively minimal loading during the stance phase [14, 35, 36]. During walking, the MCL force patterns were variable across four different studies [14, 26, 35, 36] (Fig 3C). Shao et al. [35] found high fluctuating tension with two peaks at heel-strike and mid-swing; Frigo et al. [14] and Hu et al. [26] reported higher forces in the swing phase compared with the stance phase; whilst Shelburne et al. [36] calculated a relatively low constant force of about 20N throughout the gait cycle. The LCL forces were similarly variable. However, three studies [28, 35, 36] (Fig 3D) generally show all loading occurring in the stance phase. Frigo et al. [14] and Hu et al. [26] again differed from the other models, suggesting that loading occurs throughout the stance and swing phase.

*Squatting (active/passive)*. Three studies reported ligament loads during squatting [28, 31, 37] (Fig 4). Shelburne et al. [37] generally showed minimal activity across all the ligaments (Fig 4A, 4C and 4D) except for the PCL, which increased from 35° of knee flexion up to about 600N at full knee flexion (Fig 4B). Bersini et al. [31], via modelling the passive squatting, also showed increased PCL force with increasing knee flexion (Fig 4B) and a similar load profile for MCL (Fig 4C). An inverse loading profile, which decreased with increasing flexion, was reported for the ACL (Fig 4A) and LCL (Fig 4D). The LCL experienced relatively low loads; these loads were fairly constant for Shelburne et al. [37] at 0N and Kang et al. [28] at 90N (Fig 4D) modelling the active squatting.

*Knee flexion (passive)*. The ACL and PCL knee flexion forces of a musculoskeletal model under passive knee flexion are only calculated and reported by two studies [30, 38] (Fig 5). Shelburne et al. [38] showed that the ACL generally experiences a high force (~100N) at full extension, which reduces to nil by about 60° of flexion [38] (Fig 5A). In contrast, the PCL experiences no force from 0° to 40° of flexion, followed by a steadily increasing force with increasing knee flexion [38] (Fig 5B). Kia et al.’s model showed comparatively low forces with minimal variation throughout the ACL and PCL flexion range [30] (Fig 5B and 5A).

*Anterior-posterior drawer test (passive)*. Of all 25 studies in this systematic review, only one study calculated the ACL and PCL force pattern during the passive tibial anterior-posterior drawer test [31]. Bersini et al. [31] found that the force required to draw the tibia forward (equivalent to 5mm displacement) was higher (328N) when the knee was extended than when it was flexed (226N). In contrast, the force required to draw the tibia backward (again, equivalent to 5mm displacement) was higher (298N) when the knee was flexed than when it was extended (138N).

During anterior drawing, they showed approximately 400N (at 0°) and 50N (at 90°) of force experienced in the ACL, and almost zero forces at 0° and 90° of flexion in the PCL. The posterior drawer, instead, produced a significant load on PCL, with approximately 110N (at 0°) and relatively higher force values of 319N (at 90°), while in the ACL, zero forces (at 0°) and 75N (at 90°) was reported. It is clear that increasing the flexion angle reduces the ACL force and decreases the PCL force, which is consistent with the results presented in Figs 3 and 5 above.

#### Predicted strain/elongation

**Fig 6:**
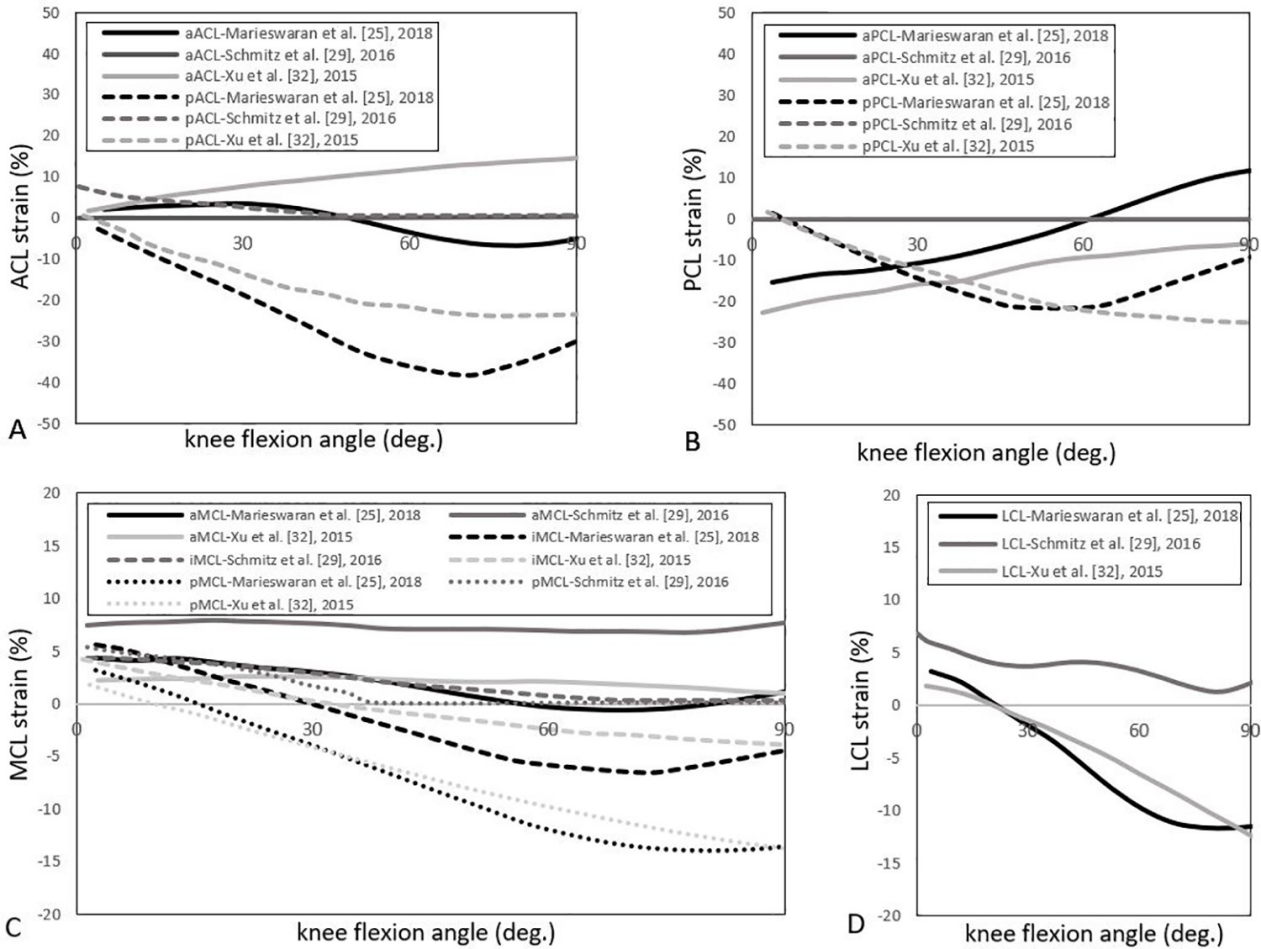
Reported ACL, PCL, MCL and LCL percentage of strain vs knee flexion angle during passive knee flexion in 3 different studies [25, 29, 32]. Curves represent the trends of ligament strains (in per cent) within different bundles of the ACL (A), PCL (B), MCL (C), and LCL (D) in 0° to 90° of knee flexion along with the passive flexion movement. The MCL and LCL strains are presented in the -20 to +20% range to highlight the trend more clearly.

Ligament strain data was reported in five studies [24, 25, 29, 32, 34]. These studies either refer to a reference length [24, 32, 34] or a slack length [25, 29] extracted from the literature. A reference length is the ligament length in a defined body posture (e.g., full extension in passive movements or heel-strike in walking), whereas the slack length refers to the length at which the ligament is slack. Three of these studies separated the cruciate ligaments into their respective bundles and investigated the passive knee motion using OpenSim [25, 29, 32]. Fig 6 shows the strain values for the cruciate and collateral ligaments over 90° of passive flexion. The strain magnitudes of the different ligament bundles were found to exhibit different patterns throughout flexion (Fig 6), with considerably higher strains reported for the ACL and PCL ligament bundles (Fig 6A and 6B). Also, MCL Strain values within different bundles of MCL displayed considerable variation (Fig 6C). Although most of the MCL bundles had declining strain patterns, anterior bundles of the MCL are tensioned almost constantly during the flexion, while intermediate bundles of the MCL showed small strain values than the posterior bundles. LCL strain values were in a similar range and pattern to that of MCL bundles (Fig 6D), having a slow declining strains trend throughout the range of flexion. However, Schmitz et al. [29] suggested positive LCL strain values with a maximum at full extension.

Two other studies also reported the ligament strain patterns: Kar et al. [34] reported active ACL strains and forces under stop-jump activities, with strains reported between 6–10%. Barzan et al. [24] reported cruciate and collateral ligament strains calculated based on optimisation routines within three different ligament models and validated using MRI scans of paediatric subjects at various flexion angles.

**Fig 7:**
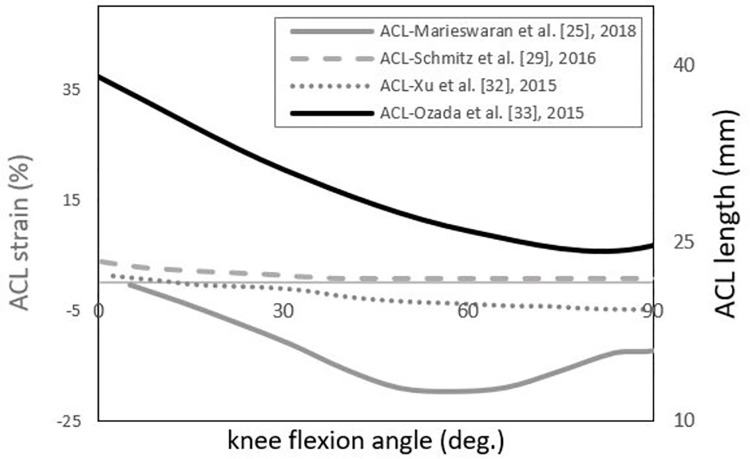
The reported percentage of ACL strain and length in 4 different studies [25, 29, 32, 33]. Grey curves represent the trends of ACL strains (in per cent), and the black curve represents the ACL length (in millimetre) in 0° to 90° of knee flexion along with the passive flexion movement. To highlight the trends more clearly, ACL strain values within the anterior (aACL) and posterior (pACL) bundles of three studies were averaged and presented here.

In two studies, ligament length/elongation was reported by measuring the ligament’s distance between origin and insertion points [22, 33]. In those studies, the ligament length at each knee joint pose was compared to quantify the relative length change throughout the activity. The length change of knee model ligaments was investigated under passive knee motions by Ozada et al. [33]. Their results demonstrated that by increasing the flexion angles from 0° to 90°, the ACL shortened, consistent with the average ACL strain values within anterior and posterior bundles, reported above [25, 29, 32](Fig 7). Considering the contact between the tibia and femur, Smale et al. [22] used extensive optimisation methods [44] to ensure realistic joint contact behaviour, reporting knee ligament lengths for a side cut task. Their models covered a wide range of possible ligament lengths.

## Discussion

### Analysis of the model predictions

**Fig 8:**
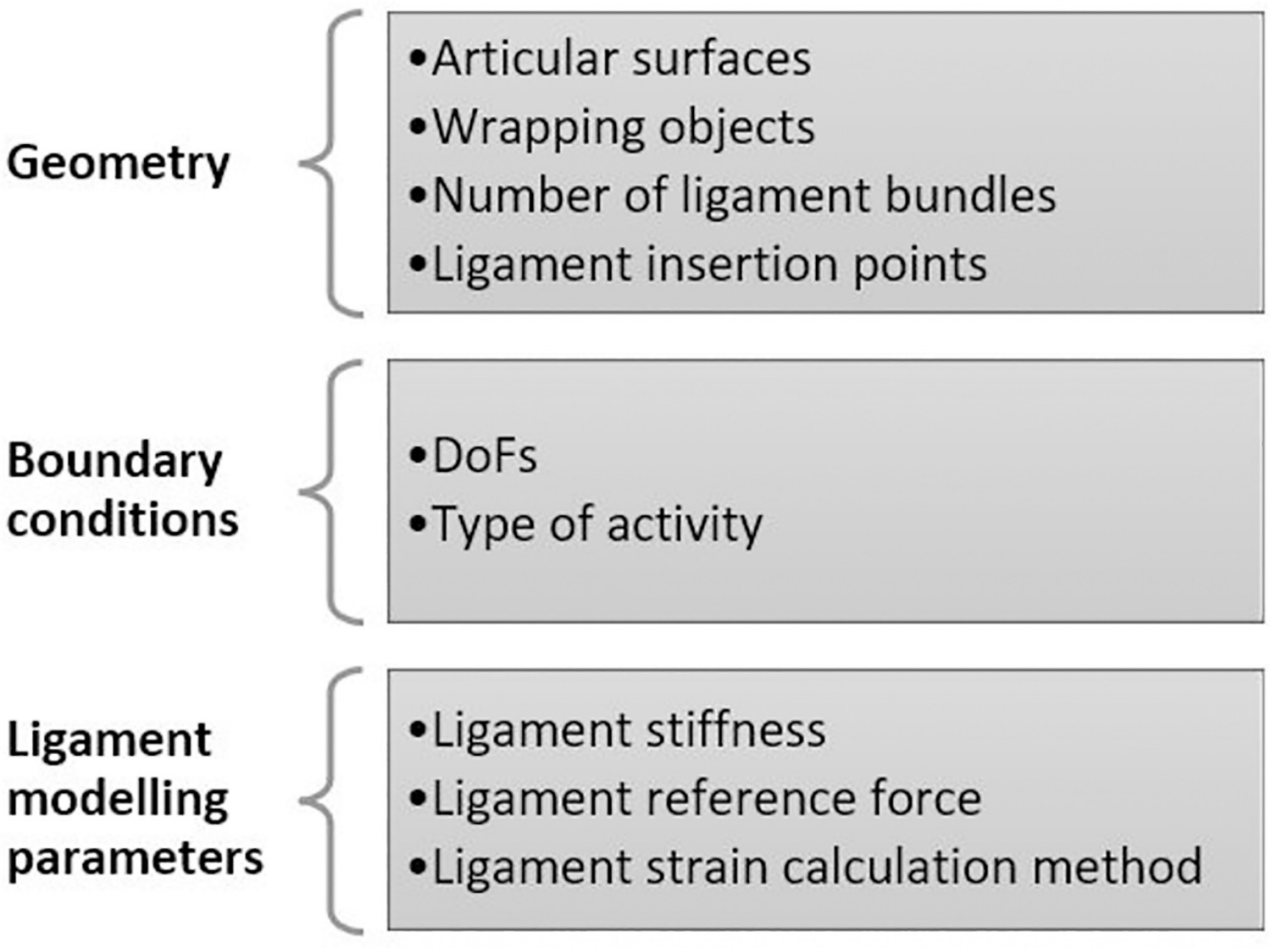
Categorised parameters of musculoskeletal knee model.

Various modelling techniques under different boundary conditions have been used to predict knee joint kinematics and ligament mechanics. Because of these differences, there is considerable variability in the resulting ligament mechanics. Geometric input, boundary conditions, and ligament modelling parameters are used to define the unique characteristics of each specific musculoskeletal model (Fig 8). All these parameters have, to varying degrees, been found to affect the predicted ligament mechanics behaviour. Below is a discussion relating to each of these parameters.

#### Effect of geometry on the predicted ligament mechanics

*Articular surfaces*. The inclusion of cartilage and the menisci, as opposed to rigid body contact, would ensure more natural contact forces and pressure distributions [45–49]. In musculoskeletal knee models, the menisci also serve as kinematics constraints in the posterior and medial-lateral direction [25, 50], improving forward dynamics predictions by minimising numerical instabilities and allowing a more natural movement to be mimicked [51] hence loading the surrounding soft-tissue more realistically. A relatively small number of studies have modelled the cartilage and menisci [18, 25, 26, 30]. However, studies using alternative modelling techniques, such as finite element modelling studies [52–54], have also highlighted the importance of considering these articular surfaces.

One concern associated with modelling the cartilage and menisci is determining the appropriate material properties for the specific model [55, 56]. Some guidance for material property selection is given in the literature [56, 57]. However, the ranges in property values tend to be very wide, providing very rough guidance, given the number of other parameters that also need to be considered in building a model.

*Wrapping objects*. Several papers modelled the collateral ligaments using wrapping objects [22–25, 28, 29, 33]. This produces a more natural elongation of the ligament, forcing it to glide over the condyles rather than allowing them to pass through the condyles as linear elements. This benefits from stabilising the joint by incorporating the knee capsule’s encapsulating effect into the model. Although no study has compared modelling the ligaments as linear vs curvilinear, it can be deduced from the literature that this is an important parameter. For instance, Hu et al. [26] modelled the collateral ligaments (ligaments that normally stabilise the varus-valgus motion) as straight lines. Their results (Fig 3) showed a higher range of forces within LCL and MCL during the swing phase of walking (implying greater movement in the varus-valgus and mediolateral direction) compared to the forces experienced by Shelburne et al.’s [58] curvilinear modelled ligaments with wrapping objects. This difference indicates the insufficient constraint and instability present at the joint in the absence of wrapping objects.

*Ligament bundles*. Ligaments with larger footprints can be modelled as ligament bundles. Adding additional elements to the ligament bundle captures the physical dimensions of the ligament better than simply changing the material properties of a single element. The use of multiple-bundle models is recommended to investigate ligament mechanical properties as this better captures the range of variation across the attachment site of the ligaments [59].

The MCL spans over a wider width than the LCL [60]. Therefore, the respective number of line elements and width of the modelled MCL should reflect this to reproduce the natural movement and stability of the joint. Some studies [20, 22, 24, 26, 33] have modelled the MCL and LCL as equally wide and thus equally constraining. For instance, this may contribute to the larger range of motions observed in the varus-valgus and mediolateral directions in Hu et al. [26], as seen in the swing phase of Fig 3C.

Some models [35–38] have included both superficial and deep bundles of the MCL (Table 4), which better mimic the natural anatomy. Whether they affect the results output by the model cannot be confirmed, as no study has made this direct comparison. However, it is likely that including all recognised bundles ensures the appropriate stability is provided to the joint.

The effect of including accessory ligaments (e.g. CAP- posterior capsule ligament) can be seen in models such as Shelburne et al. [37], who found that including the CAPs resulted in lower MCL and LCL forces (Fig 4C and 4D) compared to other models [31]. Shelburne et al. [37] also reported higher PCL and lower ACL forces during squatting (Fig 4A and 4B) compared with Bersini et al. [31], which is possibly due to the inclusion of the CAP as it provides an extra constraint in the posterior direction. CAPs have been shown to limit varus laxity, internal tibial rotation, external rotation, and posterior translation [61], which helps to explain the above results.

*Ligament insertion points*. The appropriate positioning of ligament insertion points has been shown to be important in a range of sensitivity studies in the literature [18, 23, 36]. Ideally, patient-specific scans are used to determine ligament insertion points [5, 9] accurately. Otherwise, generic models can be used in conjunction with existing sensitivity analyses to approximate an appropriate insertion point [36].

Sensitivity analyses in the literature reveal that the insertion points of all ligaments have some impact on the kinematics and contact forces in the knee [18, 23]. For example, one study [23] found the active kinematics of the knee joint to be sensitive to the LER attachment site. Their sensitivity study found a postero-proximal femoral attachment site to behave more similarly to an intact knee. Vignos et al. [18] modelled the ACL by 500 virtual bundles in a sensitivity analysis of a simulated walking, which could assess the effect of different locations of ACL graft on the experienced ACL force. They found that ACL forces were sensitive to vertical graft angle during walking; A more vertical ACL graft induces greater anterior tibial translation, ACL loading, and posterior migration of contact points on the tibial plateaus.

Recent studies, such as Vignos et al. [18], Bersini et al. [31], Kang et al. [28] and Barzan et al. [24], use MR images were used to extract the exact ligament insertion points or use the direct measurement from dissected cadavers [30].

### Effect of boundary conditions on the predicted ligament mechanics

**Fig 9:**
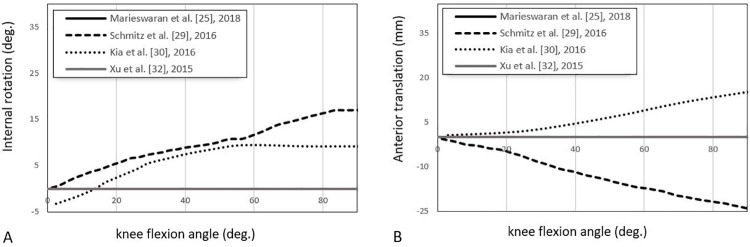
Reported kinematics of the knee joint during passive knee flexion [25, 29, 30, 32]. Curves represent the kinematic of the knee joint in forms of internal tibial rotation (in degrees) (A) and anterior tibial translation (in millimetres) (B) in 0° to 90° of knee flexion along with the passive flexion movement.

*DoFs*. Five studies [25, 29, 30, 32, 38] assessed ligament mechanics during passive knee flexion/extension tests (Figs 5 and 6). As summarised in Table 6, each applied different boundary conditions as they tried to simulate the tibia’s passive movement relative to the femur. The variable knee kinematics results throughout the range of knee flexion indicate the effect of different degrees of freedom on the model performance [25, 29, 30, 32, 38] (Fig 9).

**Table 6:** Comparison of the tibiofemoral joint boundary conditions among five studies [25, 29, 30, 32, 38] which reported the knee kinematics during the *passive knee flexion* test.

Study	Locked/Unlocked DoFs						Applied load	Measured Ligament force/strain values				
	Flexion-Extension	Internal-External tibial rotation	Abduction-Adduction	Anterior-Posterior translation	Medial-Lateral translation	Proximal-Distal translation	force/torque	Ɛ/N	ACL	PCL	MCL	LCL
Marieswaran et al. [25], 2018	unlocked	locked	locked	locked	locked	locked	Force: 0 N Torque: 0 Nm Passive knee flexion (0–120°) was simulated by fixing the knee at different flexion angles.	Ɛ	**aACL**: *(Pre-tension strain*: *0% Ɛ)* 0–45°: 0–4% Ɛ 45–90°: -ve Ɛ	**aPCL**: *(Pre-tension strain*: *-15% Ɛ)* 0–60°: -ve Ɛ 60–90°: 0–10% Ɛ	*(Pre-tension strain*: *5% Ɛ)* 0–30°: 0–5% Ɛ 30–90°: -ve Ɛ	*(Pre-tension strain*: *5% Ɛ)* 0–20°: 0–5% Ɛ 20–90°: -ve Ɛ
									**pACL**: *(Pre-tension strain*: *0% Ɛ)* 0–90: -ve Ɛ	*(Pre-tension strain*: *0% Ɛ)* 0–90: -ve Ɛ		
Schmitz et al. [29], 2016	unlocked	unlocked	locked	unlocked	locked	locked	Force: 0 N Torque: 0 Nm assive knee flexion (0–90°) was defined as a prescribed function of time in the model	Ɛ	**aACL**: *(No Pre-tension)* all 0% Ɛ	**aPCL**: *(Pre-tension strain*: *0% Ɛ)* all 0% Ɛ	*(Pre-tension strain*: *8% Ɛ)* 0–90°: 8% Ɛ	*(Pre-tension strain*: *8% Ɛ)* 0–90°: 3–8% Ɛ
									**pACL**: *(Pre-tension strain*: *8% Ɛ)* 0–50°: 0–8% Ɛ 0–90°: all 0% Ɛ			
										**pPCL**:*(Pre-tension strain*: *0% Ɛ)* 0–90: 0% Ɛ		
Xu et al. [32], 2015	unlocked	locked	locked	locked	locked	locked	Force: 0 N Torque: 0 Nm Passive knee flexion (0–120°) was simulated by fixing the knee at different flexion angles	Ɛ	**aACL**: *Pre-tension strain*: *0% Ɛ)* 0–90°: 0–12% Ɛ **pACL**:	**aPCL**: *(Pre-tension strain*: *0% Ɛ)* -ve Ɛ	*(Pre-tension strain*: *3% Ɛ)* 0–20°: 0–3% Ɛ 20–90°: -ve Ɛ	*(Pre-tension strain*: *3% Ɛ)* 0–20°: 0–3% Ɛ 20–90°: -ve Ɛ
									*(Pre-tension strain*: *0% Ɛ)* -ve Ɛ	**pPCL**: *(Pre-tension strain*: *0% Ɛ)* -ve Ɛ		
Kia et al. [30], 2016	unlocked	unlocked	unlocked	unlocked	unlocked	unlocked	Force: 10 N Torque: 0 Nm Passive knee flexion (0–130°) was simulated by rotating the femur about the knee flexion axis while applying 10N of compression force	N	*(Pre-tension force*: *20N)* 0–90°: 0-20N force	*(Pre-tension force*: *10N)* 0–90°: 10N force	*(Pre-tension force*: *5N)* 0–90°: 5-10N force	*(Pre-tension force*: *15N)* 0–20°: 0-15N force 20–90°: 0N
	
Shelburne et al. [38], 1997	unlocked			locked		locked	Force: 320 N Torque: 0 Nm Passive knee flexion (0–90°) was simulated by rotating the femur about the knee flexion axis while applying 320N of quadriceps pull force	N	*(Pre-tension force*: *150N)* 0–90°: 10-150N force	*(Pre-tension force*: *0N)* 0–35°: 0N 35–80°: 0-70N force	NR	NR

Table 6 summarises the available degrees of freedom for each model and the respective ligament tensions. Marieswaran et al.’s [25] and Xu et al.’s [32] models both constrained all DoFs except for the flexion-extension axis. These highly constrained models result in the presence of low tension in the aACL and aPCL, with all other ligaments producing negative strains. In practical terms, negative strains indicate laxity in those soft tissues throughout the full range of motion (ROM). In contrast, Kia et al.’s model [30], which allowed the full 6-DoFs, experienced some small forces in all ligaments during passive flexion for most of the ROM.

In general, we know that the collateral ligaments experience higher tensions when there is an increase in activity in the adduction/abduction, int/external tibial rotation, or medial/lateral translation directions. Looking at Fig 9, Schmitz et al. [29] and Kia et al. [30] both had increased internal rotations and thus should expect to see increased collateral ligament tensions. Table 6 verifies this assumption; both models report strains of up to 8% or up to 10N of force in the collaterals.

Similarly, increased anterior-posterior tibial translations lead to an increase in the tensions in the cruciate ligaments. For example, in Fig 9B, Kia et al. [15] experienced increased anterior translation as the knee flexed as opposed to the increased posterior translation reported by Schmitz et al. [29], which locked three extra DoFs. Therefore, we expect the ACL and the two collaterals to undergo tension, which is verified in Table 6.

In another study, Smale et al. [22] compared different knee models with either three or 6-DoFs. The generic 6-DoFs model experienced a greater range of movement and ligament length changes. However, an MRI-based model with 6-Dof in the same study showed lower length change when validated against quasi-static MR images [22]. The authors hypothesised that the ligament lengths were too short, leading to an over-constrained joint. Also, the MRI based knee model was found to be very responsive to the kinematics and ligament lengths of highly dynamic tasks.

**Fig 10:**
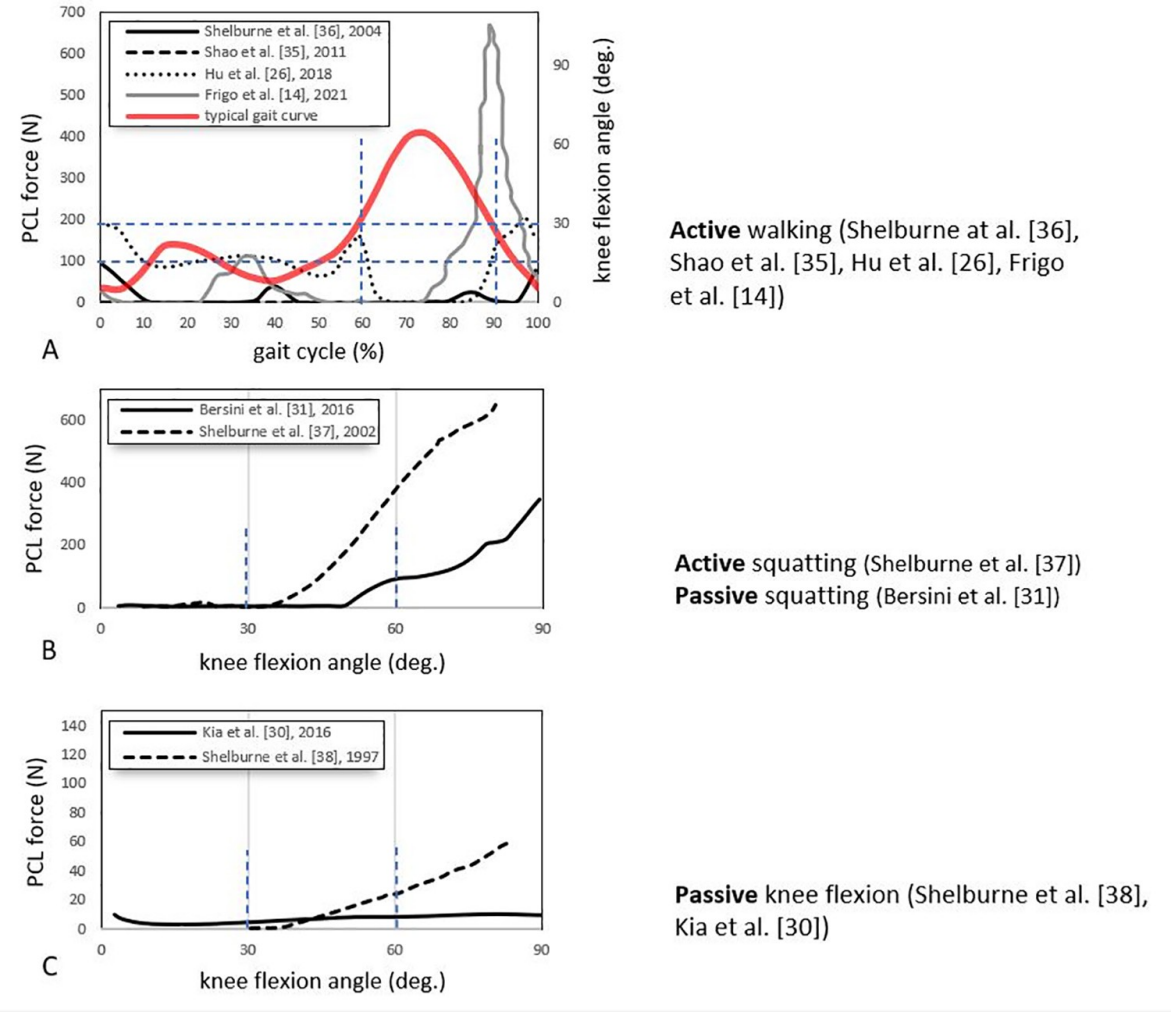
Comparison of reported PCL forces vs knee flexion angle during walking, squatting, and passive knee flexion in different studies [14, 26, 30, 31, 35–38]. Curves represent the trends of PCL forces (in Newton) during active walking (A), passive and active squatting (B), and passive knee flexion (C) in 0° to 90° of flexion. As a typical gait curve, the red curve represents the knee flexion angles vs the percentage of the gait cycle. Blue dashed lines are used to determine the corresponding flexion angle.

*Type of activity*. Load bearing activities that generate a reaction force are generally referred to as closed chain activities; open-chain activities typically do not generate a reaction force and include the swing phase in walking and passive motions. An example of the difference can be seen in the cruciate ligament forces during active (squatting) (Fig 4) and passive (knee flexion) activities (Fig 5). They follow a similar pattern, but the magnitude of the forces is significantly higher for the active results. This difference is most likely due to the closed-chain, load-bearing nature of active squatting [30, 31, 37, 38] (Fig 10B and 10C).

The difference between open- and closed-chain activities is also highlighted when comparing the PCL force during active walking (Fig 10A) with those reported by Shelburne et al. [37] during squatting (Fig 10B). Although both are active tasks, the PCL force drops to nil during the swing phase of the gait cycle, which is equivalent to 30°-60° of flexion (see red gait curve in Fig 10A); for active squatting, in this same flexion range, the PCL load is monotonically increasing. This is because the swing phase of active walking is open-chain, exerting a minimal force on the ligament. As a result, the passive knee flexion curve (Fig 10C) aligns better with the swing phase of the active walking curves (i.e., 30°-60° of flexion) since it is also an open-chain configuration.

Notably, there is a greater agreement concerning the swing phase in the active walking figure, with three studies all showing no load in the PCL (Fig 10A). In contrast, the stance phase of active walking (0–60° of knee flexion in Fig 10A) shows a large amount of variation. This indicates that joint load distribution during closed-chain activities, such as the stance-phase of walking, is more sensitive to various patient-specific parameters [62, 63]. In contrast, open-chain activities such as the swing phase of walking allow relaxation of the surrounding soft tissues and the joint mechanical behaviour relies more on any external forces and moments for passive motion [64] or momentum in active motion [65].

During the stance phase of active walking (0–60%), the knee flexion angle (Fig 10A, red curve) generally remains below 30°. Shelbourne et al. [36] and Shao et al. [35] both agree that there is minimal loading in the PCL throughout much of the stance phase, which agrees well with the results for this flexion range during active squatting (Fig 10B). As discussed below, the higher loads in Hu et al. [12] can be attributed to variations in ligament properties.

### The effect of ligament modelling parameters on predicted ligament mechanics

*Ligament stiffness*. The ligament stiffness values used in the various studies may explain some of the differences observed in ligament forces (Fig 3B) between the multiple studies. In Hu et al.’s model [26], the referencing regarding ligament stiffness values is unclear; for instance, the PCL stiffness values have a possible range of 1500-9000N (Table 7) compared to values of 1900-2600N in the Shelburne et al. model’s [36] and Shao et al.’s model [35]. This possible discrepancy may explain the differences between ligament stiffness values.

**Table 7:** ACL/PCL stiffness values from active models of walking and passive models of knee flexion.

Study	Motion	ACL		PCL		MCL		LCL	
		Stiffness	Peak Force	Stiffness	Peak Force	Stiffness	Peak Force	Stiffness	Peak Force
**Shelburne et al. [36]**	Active walking	1500-1600N	300N	1900-2600N	90N	2500-4500N	30N	2000N	120N
**Shao et al. [35]**	Active walking	1500-1600N	220N	1900-2600N	Zero	2500-4500N	120N	2000N	220N
**Hu et al. [26]**	Active walking	1500-5000N	35N	1500-9000N	200N	1000-2750N	150N	2000-4000N	160N
**Frigo et al. [14]**	Active walking	1500-1600N	200N	1900-2600N	630N	2500-4000N	120N	667N	100N
**Kia et al. [30]**	Passive knee flexion	750-850N	20N	2200-4600N	10N	3100N	10N	3300N	15N
**Shelburne et al. [38]**	Passive knee flexion	1500-1600N	150N	1900-2600N	60N	2500-4500N	NR	2000N	NR

In general, the ranges of stiffness values listed for the ligament stiffnesses are often large (Table 7). This leads to some issues with the reproducibility of the data presented in studies. It would be ideal if the final values were listed for each ligament bundle, allowing for better verification of previous findings.

*Method of calculating the ligament strain*. Many studies report a negative strain in ligaments (e.g., Fig 6). This is because their calculations are based on the relative distance between the insertion points rather than the actual strain. For instance, Xu et al. [32] and Marieswaran et al. [25] measured the relative distance between the insertion points to find the strain; this fails to account for the wrapping of the ligament around the bones and the change in strain patterns that this may create.

In contrast, Schmitz et al. [29] calculated strains based on predicted forces (i.e. indirect strain measurement) using ligament nonlinear force-strain equations [66–70]. This calculation method allows the wrapping of the ligament around the bone geometry to be incorporated in the strain calculation.

In addition, while many studies simply report the calculated value for strain (positive or negative), Schmitz et al. [29] recognised that negative strain represents the laxity of the ligament; they set a zero strain threshold to incorporate this into their model.

### Sensitivity assessment

Our review reveals the need for and importance of running a sensitivity analysis when analysing joint ligament behaviour. Sensitivity analyses are important for several reasons: (¡) they indicate the accuracy of the model in the given scenario [71, 72]; (¡¡) they provide a means of investigating a window of possible outcomes given any uncertainty about analysed parameters [71, 73]; and (¡¡¡) they can potentially provide insights into the importance of different ligament parameters on such factors as knee kinematics and forces [74, 75], which can have implications for such translational concepts as graft placement as discussed below.

Ligament loading values have been shown to be sensitive to most parameter variations in all studies [14–38]. This suggests that using patient-specific data to determine the exact ligament insertion points and develop accurate contact surface geometries will play an important role in future MSK models. The variation of the ligament sensitivity assessment techniques and results reported in the literature (Table 5) makes it difficult to determine how different modelling parameters affect the ligament results. However, investigating the sensitivity of ligament loading to changes in various modelling parameters using patient-specific models as a starting point may improve our understanding of these relationships.

### Quality of input data

Another source of error in knee ligament dynamics predictions between different models is the quality of the kinematics and/or kinetics data input into the model [76, 77]. Kinematic information is typically captured using motion capture methods or fluoroscopy. Both techniques have limitations that have an impact on the quality of data [78]. For example, motion capture methods can vary depending on the different marker sets used (i.e., The modification of the Helen Hayes markers set [79]), which can vary across different research groups. Also, the actual placement of the markers will vary in accuracy depending on the skill of the operator. Dealing with the data at the processing stage can add further potential errors and is dependent on the software, level of filtering, scaling and other software specific parameters. Kinetic input can be sourced from ground reaction forces and EMG data, each of these has similar limitations, regarding user error and researcher-specific measurement protocols.

Therefore, even in the case of two well-calibrated (and validated) models, the model predictions may not agree, as they will be dependent on operator error at several stages in the process before the modelling even occurs; and this is the main focus of this systematic review.

### Clinical application

Among the selected studies, in addition to models which examined the intact knee joint [14–17, 19–22, 24–27, 29–34, 37, 38], some considered aspects of knee injuries and surgical treatments, including ACL deficient [35, 36] and ACL-reconstruction [18], PCL-deficient [28] and LER reconstruction [23]. Those studies were developed for clinical applications to investigate the impacts of injuries and surgical treatments on the knee joint’s biomechanical functioning.

Shelburne et al. [36] aimed to predict the pattern of shear force and ligament loading in the ACL-deficient knee during walking. They found that increasing anterior tibial translation (ATT) reduced patellar tendon angle and reduced anterior tibial shear force. They suggested that while the MCL acts as the primary restraint to ATT in the ACL-deficient knee, patellar tendon angle changes can decrease the knee’s total anterior shear force. Shao et al. [35] investigated the influence of increasing tibial slope on ligament loading and anterior tibial translation in healthy and ACL-deficient knees during gait. Their model results gave a more in-depth insight into how the patients adapted their gait following the ACL deficiency. They found an increase in ATT throughout the stance phase for the ACL-deficient knee compared to the healthy knee. They also reported that the primary passive restraint of anterior shear force was the ACL in the healthy knee, and the MCL was the primary passive restraint to anterior shear force in the ACL-deficient knee. They also showed that the knee flexors were used as active restraint to help balance anterior shear force in the ACL-deficient knee. Based on their model results, they anticipated that increasing the tibial slope would increase the resulting ATT and ligament forces in both healthy and ACL-deficient knees.

Vignos et al. [18] explored the relationship of ACL graft angle with tunnel location with tibiofemoral kinematics in patients with ACLR. They found that post-operative cartilage loading is sensitive to the graft angle. Their results suggest that even a slight change of the graft tunnel placement leads to a small deviation from the anatomic ACL angle and an increase in knee osteoarthritis risk after ACLR.

Kang et al. [28] investigated the effect of PCL deficiency on the posterolateral corner structure forces, including the LCL, Popliteus tendon (PT), and Popliteofibular ligament (PFL), and tibiofemoral and patellofemoral contact forces under dynamic-loading conditions. They showed that PCL deficiency affects the variability of force on the popliteus tendon in dynamic-loading conditions, suggesting potential degeneration of the patellofemoral joint resulting from high flexion dynamic activity.

Blache et al. [23] assessed the effect of different LER graft tunnel locations on graft tensioning and altered knee joint kinematics. By simulating a pivot-shift test, they revealed the importance of the LER in ACL rupture patients. They suggest a postero-proximal femoral LER attachment site in LER surgery since it provides the desired behaviour during physiological knee flexion.

Kim et al. [15] investigated the effect of hip abductor weakness on the altered lower extremity joint moments and kinematics, which leads to an increased risk of ACL injury during single-leg landing. They suggest that subject-specific musculoskeletal simulations estimating ACL loading can help clinicians predict potential ACL injuries during dynamic movements.

In a similar study, Moon et al. [16], using musculoskeletal modelling techniques, found that the ACL load, following hamstring fatigue, did not show statistically significant differences; instead, there was a significant reduction in ACL load after quadriceps fatigue. This reduction after quadriceps fatigue reconfirms that the quadriceps is the major muscle group causing ACL injuries by reducing the extension and adduction moment of the knee joint and thereby increasing the ACL load.

Moon et al. [27] evaluated the effectiveness of wearing commercialised sports knee braces and sleeves on knee kinematics, kinetics, and ACL force during drop jumps using musculoskeletal modelling analysis. They found that knee braces and sleeves reduced flexion and abduction movement and adduction moment but did not reduce the knee joint shear force, internal rotation moment, or the ACL force. They suggested that a sports knee brace that controls the knee joint’s shear force and internal rotation moment needs to be developed to prevent ACL injuries during high impact activities.

### Recommendations

Based on the results of this systematic review, we recommend attention be given to the following stages of the modelling process:

#### Model definitions

The ideal approach for developing accurate models is to make them patient-specific by using CT and MR images to create precise geometries and identify other soft tissues and landmarks, such as the menisci, cartilage, and ligament insertion points. The authors recognise that the development of a generic model which can be scaled based on patient-specific anthropometric measurements might provide a more translatable clinical tool. However, this review has highlighted a general lack of consensus between ligament loading results derived from generic models. This suggests that a deeper understanding of ligament mechanics may be required before a clinically useful generic model can be developed. This deeper understanding can be reached through more patient-specific musculoskeletal models, validated with patient test data.

Additional modelling considerations that the authors believe will bring the musculoskeletal simulations closer to a realistic model are the use of wrapping objects to define the curvilinear path of the soft tissue around the bony surfaces; and the accurate modelling of ligament width using ligament bundles. Wrapping surfaces are important as they force the ligaments to wrap around bony surfaces, resulting in more realistic load and strain calculations. Ligament bundles should reflect their natural geometry and dimensions; for example, the greater width of the MCL insertion points relative to those of the LCL should be considered in developing the musculoskeletal model.

#### Boundary conditions

This review has revealed that modifying the degrees of freedom in a joint greatly impacts loading in the joint and surrounding soft tissues. The results suggest that over-constraining the joint can lead to artificially high ligament loading and range of motion [80]. The authors believe that, to determine the impact of knee kinematics on ligament loading, models need to include 6 degrees of freedom and provide realistic limitations to extreme movements that replicate a realistic range of motion.

#### Sensitivity analysis

We recommend that all models run a sensitivity analysis to assess the effect of ligament parameters on the ligament loading and joint mechanical behaviour (kinematics/kinetics).

In inverse dynamics simulations, the ligaments have no impact on the kinematics, and therefore the calculated ligament strains/forces depend on the accuracy of the material properties, recorded knee kinematics and location of ligament insertion sites. In forward dynamics simulations, results are dependent on many additional parameters, including contact surface geometry, boundary conditions, muscle definitions and force patterns. An improved understanding of how changes in all these parameters affect the performance of both inverse and forward dynamics models is important for translating this technology into a useful clinical tool.

#### Validation of the model outcomes

Vicceconti et al. [81] recommended a set of minimal requirements for validating a finite element model. They suggested that sensitivity analyses and validation against a set of controlled experiments be undertaken and reported when publishing a modelling study. We recommend that a similar standardised validation framework be developed for passive musculoskeletal simulations. A series of specific movements under specific boundary conditions should be applied to the musculoskeletal model and be compared against a standard dataset; ideally, all publications would report these motions and the respective/corresponding forces, torques or strains within soft tissue.

Knee motion can be considered to be comprised of two parts; a passive envelope, where the stiffness of the ligaments does not play a significant role, and an extreme envelope, where the stiffness of the ligaments is critical for knee function [82]. Within the passive envelope, the kinematics of the joint is dependent on a large range of parameters, and any loading or laxity of ligaments is difficult to assess. If a ligament is lax, the mechanical behaviour of that ligament cannot be evaluated, making validation of the ligament performance impossible.

Therefore, a standard dataset should be developed using cadaveric tissue for the validation of passive activities. The knee should be forced into extreme internal/external rotation with a predetermined load to ensure tension in targeted ligaments. This approach aims to develop a series of datasets where the ligament performance is dependent on parameters that can be controlled in the musculoskeletal model. Several studies have already used this idea (e.g. Blankevoort et al. [82] and Neri et al. [83]). A standard joint coordinate system (JCS), such as the Grood and Suntay [84, 85], should also be adopted in combination with these activities. In this way, predicted forces and movements could also be compared across different studies, unlike the current status of the literature.

For active simulations, gait data can be used to validate the model. Contact forces in the joint and tension in the ligaments and other soft tissues are more evident during load-bearing activities. However, the more parameters measured and replicated in the model simulation, the more valid the model is. Examples of the types of data that can be used to validate active motions in the knee are:

➢ Kinematics: In vivo accelerometers (e.g., IMUs)/dynamometers/dynamic MRI/fluoroscopy can all be used to validate the predicted joint motion of a musculoskeletal model.
➢ Kinetics: Force plates and *in vivo* EMG data can be used to compare the predicted muscle forces with those measured in active motion.

Additional resources that can help with the validation of kinetic model outputs (i.e., joint reaction forces) include those made available on such websites as www.orthoload.com, which provides free access to experimentally measured forces using instrumented implants. The "Grand Challenge Competition” database [86]" is another useful source to predict In Vivo Knee Loads based on a series of comprehensive publicly available in vivo data sets for evaluating musculoskeletal model predictions of contact and muscle forces in the knee joint. These resources can provide a valuable check on model validity.

### Limitations

The studies selected for this systematic review were limited only to those reporting ligament loading data in a musculoskeletal knee model. Therefore, several musculoskeletal studies modelling the knee joint were excluded accordingly. For example, four studies [74, 87–89] considered the effect of their modelled ligaments on the knee kinematics but did not report the ligament loading, excluding them from this review. By extending the range of studies considered to those who assessed the knee joint kinematics and kinetics, without reporting the ligament mechanics, it might be possible to better evaluate the effect of passive ligament performance on the knee functions during inverse or forward simulations.

Only a few activities were investigated in the selected database, i.e., walking, squatting, and passive knee flexion. There was insufficient data to draw any meaningful conclusions about ligament loading during each type of activity.

The limited number of studies, wide variety of simulation techniques used, and the model’s apparent sensitivity to a large number of interdependent parameters means that a comparison between the loading results did not reveal any discernible trends for the predicted ligaments loading.

Moreover, the limited number of studies specific to this systemic review criteria have frequently been conducted by members of the same research groups, likely leading to an increased risk of bias towards similar types of methodologies and results.

## Conclusion

This systematic review has looked at the current state of research regarding the loading of ligaments supporting the knee. The ligaments have a critical role in providing the knee joint stability during various physiological activities. With the help of articulating surfaces, muscle forces, and other soft tissue constraints such as the joint capsule, these passive connective structures support the knee joint’s correct biomechanical function.

By merging all reported data on knee ligament mechanics derived from musculoskeletal modelling, this systematic review revealed that there is currently a lack of consensus on the mechanics of the ligaments of the knee during various load-bearing and passive activities. This review has revealed that the lack of consensus is likely due to a lack of consistency in the model definition and the lack of a uniform system for validating the models. To improve the accuracy and robustness of the model’s predicted outcomes, the sensitivity of the models to critical ligament parameters needs to be further investigated.

Despite the current lack of consensus, this review has also highlighted the potential of developing translational tools using musculoskeletal modelling. Refining the approach using accurate model design and sensitivity assessment of the model outcomes and appropriate validation methods may result in the development of clinical tools that could be used for patient-specific treatments.

## Supporting information

S1 Checklist PRISMA checklist. (DOC)
